# Omentin-1 Modulates Porcine Endometrial Steroidogenesis and Tissue Remodelling During Early Pregnancy and the Oestrous Cycle

**DOI:** 10.3390/ijms27177731

**Published:** 2026-08-28

**Authors:** Oguzhan Koker, Grzegorz Kopij, Marlena Gudelska, Katarzyna Kisielewska, Kamil Dobrzyn, Ewa Zaobidna, Anna Nynca, Tadeusz Kaminski, Nina Smolinska, Marta Kiezun

**Affiliations:** 1 Department of Animal Anatomy and Physiology, Faculty of Biology and Biotechnology, University of Warmia and Mazury in Olsztyn, 10-719 Olsztyn, Poland; oguzhan.koker@doktorant.uwm.edu.pl (O.K.); kamil.dobrzyn@uwm.edu.pl (K.D.); anna.nynca@uwm.edu.pl (A.N.); tkam@uwm.edu.pl (T.K.); nina.smolinska@uwm.edu.pl (N.S.); 2 Doctoral School, University of Warmia and Mazury in Olsztyn, 10-719 Olsztyn, Poland; 3 InLife Institute of Animal Reproduction and Food Research, Polish Academy of Sciences, 10-683 Olsztyn, Poland; g.kopij@pan.olsztyn.pl; 4 Department of Anatomy and Histology, School of Medicine, Collegium Medicum, University of Warmia and Mazury in Olsztyn, 10-082 Olsztyn, Poland; marlena.gudelska@uwm.edu.pl (M.G.); katarzyna.kisielewska@uwm.edu.pl (K.K.); 5 Department of Biochemistry, Faculty of Biology and Biotechnology, University of Warmia and Mazury in Olsztyn, 10-719 Olsztyn, Poland; ewa.zaobidna@uwm.edu.pl

**Keywords:** omentin, embryo implantation, pregnancy, steroidogenesis, endometrium, Akt signalling pathway, apoptosis/proliferation

## Abstract

In pigs, the establishment of pregnancy depends on tightly coordinated molecular interactions between the conceptus and the maternal endometrium during the peri-implantation period. Adipokines, peptide hormones produced by adipose tissue, function as endocrine mediators linking metabolic status with uterine function. Omentin-1, a hormone belonging to the adipokines group, is hypothesized to play a potential role in regulating female reproductive functions through its influence on uterine functions. Therefore, this study investigated the effects of omentin-1 on endometrial progesterone and oestradiol secretion using radioimmunoassay, on the abundance of key steroidogenic proteins and Akt phosphorylation using Western blot, and on endometrial cell proliferation and apoptosis using flow cytometry. Results showed that its actions are characterised by promotion of progesterone-dominant conditions, selective modulation of steroidogenic pathways, activation of Akt signalling, and cell-type-specific regulation of proliferation and apoptosis. This combination of effects positions omentin-1 as a previously unrecognised regulator of endometrial adaptation and suggests that adipokines may represent an important mechanistic link between metabolic status and reproductive success.

## 1. Introduction

In pigs, the establishment of pregnancy depends on tightly coordinated molecular interactions between the conceptus and the maternal endometrium during the peri-implantation period [1]. One of the key events in the establishment of pregnancy in pigs is the first conceptus-derived oestrogen signal, which increases markedly around days 11–12 of gestation during rapid conceptus elongation and constitutes a major component of maternal recognition of pregnancy. This oestrogen directs the uterine release of prostaglandin F2α (PGF2α) into the uterine cavity, preventing luteolysis and ensuring the preservation of the corpus luteum and progesterone (P_4_) production, which are essential for embryo survival and implantation [2,3,4]. Beyond responding to steroids and signals from the ovary and embryo, the porcine uterus is an active endocrine tissue capable of locally synthesizing steroid hormones. Previous studies have shown that the endometrium and myometrium produce P_4_, oestradiol (E_2_), oestrone (E_1_), androstenedione (A_4_), and testosterone (T) during early pregnancy and the oestrous cycle, suggesting that locally produced steroids may contribute to the regulation of uterine receptivity and embryonic development [5,6,7]. E_2_ and P_4_ precisely orchestrate epithelial remodelling through adjusting proliferation/apoptosis to provide a receptive uterus for implantation [8]. Therefore, the local production of steroids indicates that uterine functions are regulated locally to some extent.

Mechanisms controlling energy balance are closely linked to reproductive regulation, together optimizing reproductive success under changing metabolic conditions [9]. Adipokines, peptide hormones produced by adipose tissue, function as endocrine mediators linking metabolic status with uterine function [10]. Many adipokines, like leptin, adiponectin, or chemerin, are expressed in both human and animal reproductive tissues [11,12,13,14,15,16]. Adipokines have been shown to regulate several reproductive processes, including follicular steroidogenesis, cell proliferation, apoptosis, and implantation [10,17,18]. Adiponectin, resistin, and chemerin have also been shown to affect uterine steroidogenesis [19,20,21,22]. Although the mechanisms of action are not fully understood, the presented data suggest that other adipokines, like omentin, may also be involved in the regulation of uterine steroid production.

Omentin-1, also called intelectin-1, a hormone belonging to the adipokine group, is hypothesized to play a potential role in regulating female reproduction through its influence on uterine functions [23]. Omentin-1 possesses a wide spectrum of physiological and therapeutic features [24]. Its insulin-sensitizing effects have been reported [25]. Based on these effects, its antidiabetic effects have been proposed (for a review, see [26]). Although the specific omentin-1 receptor remains unidentified, it has been implied that omentin-1 can activate the insulin receptor (INSR) downstream signalling pathways [24,27,28]. The expression of omentin-1 was demonstrated in the human and porcine ovaries, including granulosa and granulosa-luteinizing cells [29,30,31]. Ovarian expression of omentin-1 was regulated by insulin, gonadotropins, steroids, and IGF-1 [30,31]. On the other hand, the adipokine was found to increase insulin sensitivity of human granulosa cells and support steroidogenesis by upregulating P450_AROM_ and StAR and activating IGF-1 signalling [30]. Although ERK1/2/MAPK, PKA, and INSR-related signalling have been implicated in the ovarian actions of omentin-1, the adipokine has also been linked to PI3K/Akt signalling in reproductive and non-reproductive cell models [28,31,32]. Studies in pigs indicate Akt integrates insulin-sensitive metabolic signalling with pathways controlling cell survival, proliferation, and steroidogenic function [31,32,33,34].

Considering that uterine tissue shares steroidogenic capacity and insulin-sensitive regulatory mechanisms with the ovary, we hypothesised that omentin-1 modulates porcine endometrial steroidogenesis and the proliferation/apoptosis balance in a stage-dependent manner during early pregnancy and the oestrous cycle, with Akt signalling as a candidate signalling pathway, and thereby contributes to the regulation of endometrial function. Therefore, this study aimed to determine the effect of the adipokine on P_4_ and E_2_ synthesis and secretion. This localised hormone production relies on a coordinated enzymatic cascade. StAR mediates the transfer of cholesterol to the inner mitochondrial membrane, where P450_SCC_ (CYP11A1) catalyses its conversion to pregnenolone. 3βHSD catalyses the conversion of pregnenolone to P_4_ and other Δ5 steroids to their corresponding Δ4 products. P450_C17_ (CYP17A1), through its 17α-hydroxylase and 17,20-lyase activities, directs steroid precursors toward androgen biosynthesis, whereas P450_AROM_ (CYP19A1) catalyses the aromatization of androgens to oestrogens [35]. Therefore, we aimed to assess the dose- and stage-dependent effect of omentin-1 on the protein abundance of StAR, P450_SCC_, 3βHSD, P450_C17_, and P450_AROM_ in the endometrial tissue. Additionally, we aimed to assess the effect of omentin-1 on Akt signalling pathway activation as a candidate signalling response to omentin-1 in the porcine endometrium. This analysis was intended to determine whether omentin-1 affects Akt phosphorylation rather than to establish causal mediation of its biological effects through Akt. Another aim of the study was to evaluate the adipokine’s effects on endometrial cell proliferation and apoptosis. The omentin-1 concentrations used in the present experiments (25–100 ng/mL) were selected to encompass the range detected in porcine blood plasma and uterine luminal fluid in our preliminary measurements and concentrations previously noted in the study by Respekta et al. [36].

## 2. Results

The results of the analysis of variance (one-way ANOVA) are reported in Supplementary Tables S1–S5.

### 2.1. The Effect of Omentin-1 on P_4_ and E_2_ Secretion by the Porcine Endometrial Tissue Explants

**Figure 1:**
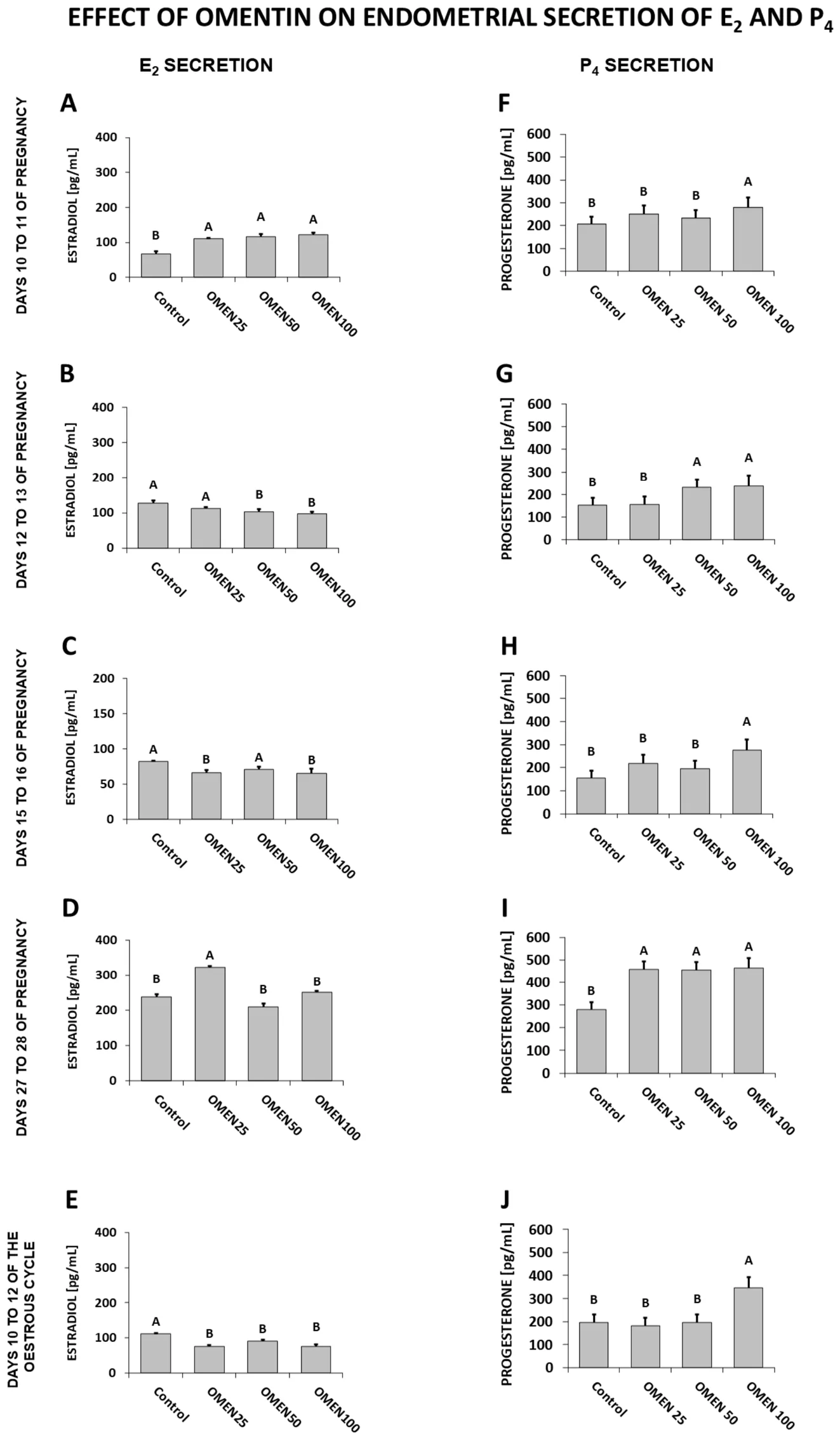
The effect of omentin-1 on the endometrial secretion of progesterone and oestradiol during the oestrous cycle and early pregnancy. The effect of omentin-1 (OMEN; 25, 50, and 100 ng/mL) on the secretion of oestradiol (E_2_; (**A**–**E**)) and progesterone (P_4_; (**F**–**J**)) by the in vitro incubated endometrial explants obtained from pigs during early pregnancy (days 10 to 11, 12 to 13, 15 to 16, and 27 to 28) and on days 10 to 12 of the oestrous cycle. Results are presented as means ± S.E.M. (*n* = 5). Statistically significant differences are presented by different letters (*p* < 0.05).

Omentin-1 increased the endometrial E2 secretion on days 10 to 11 (all doses) and days 27 to 28 (25 ng/mL) of gestation (Figure 1A,D). The secretion of E2 was decreased by omentin-1 on pregnancy days 12 to 13 (50, 100 ng/mL) and 15 to 16 (25, 100 ng/mL), and on days 10 to 12 of the oestrous cycle (all doses; Figure 1B, C, and E, respectively).

Omentin-1 (100 ng/mL) increased the endometrial P4 secretion in each examined period of pregnancy and during the oestrous cycle (Figure 1F–J), at the dose of 50 ng/mL on days 12 to 13 and 27 to 28 of gestation (Figure 1G,I), and at the dose of 25 ng/mL also on days 27 to 28 of pregnancy (Figure 1I).

### 2.2. The Effect of Omentin-1 on StAR, P450_SCC_, 3βHSD, P450_C17_, and P450_AROM_ Proteins Abundances in the Porcine Endometrial Explants

**Figure 2:**
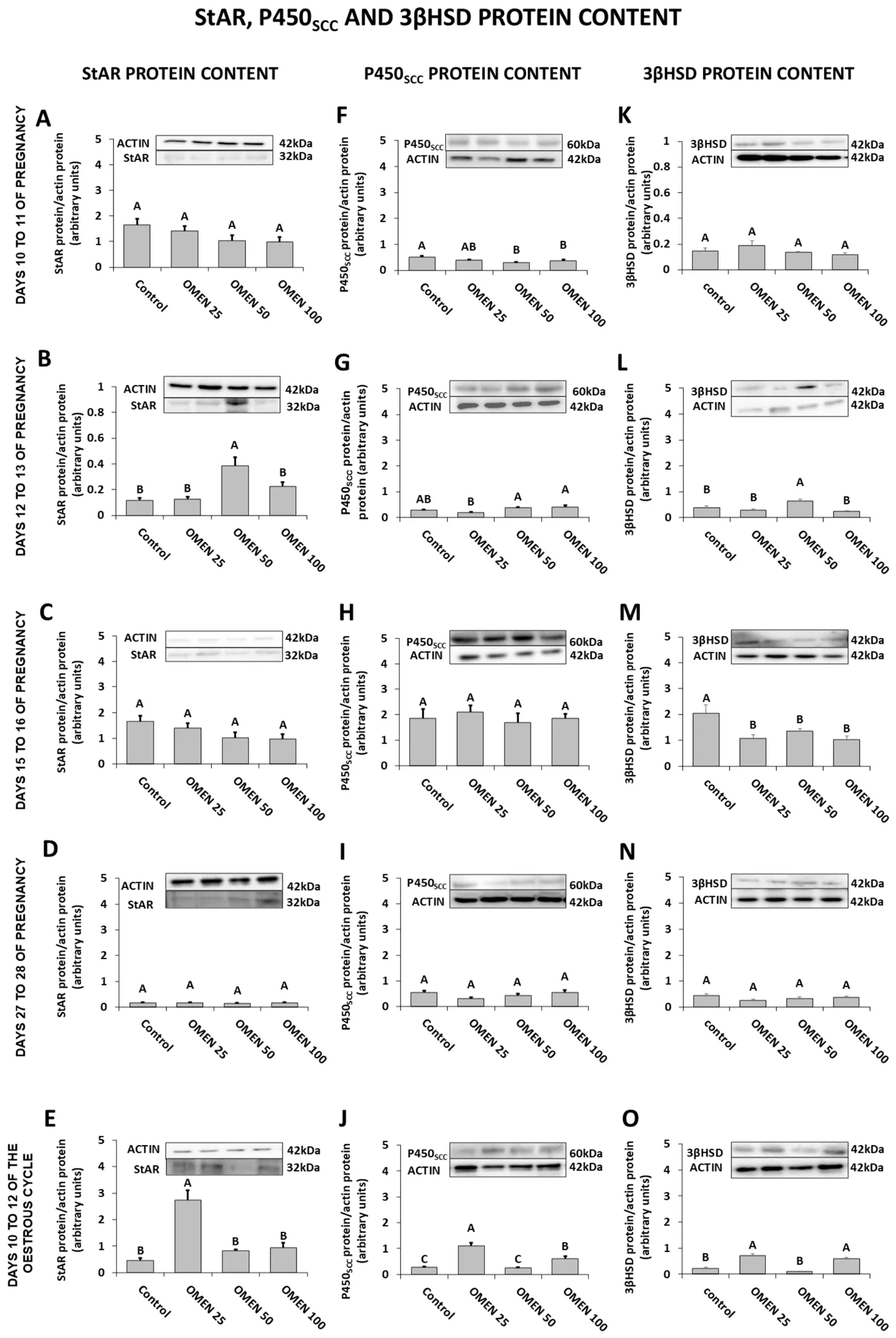
The effect of omentin-1 on the endometrial expression of StAR, cytochrome P450_SCC_, and 3βHSD during the oestrous cycle and early pregnancy. The effect of omentin-1 (OMEN; 25, 50, and 100 ng/mL) on protein abundances of steroidogenic acute regulatory protein (StAR; (**A**–**E**)), P450 side-chain cleavage enzyme (P450_SCC_; (**F**–**J**)) and 3β-hydroxysteroid dehydrogenase (3βHSD; (**K**–**O**)) in the in vitro incubated endometrial explants obtained from pigs during early pregnancy (days 10 to 11, 12 to 13, 15 to 16, and 27 to 28) and on days 10 to 12 of the oestrous cycle. Upper panels: representative immunoblots; lower panels: protein content normalized to actin protein; results (arbitrary units) are presented as means ± S.E.M. (*n* = 5). Statistically significant differences are presented by different letters (*p* < 0.05).

Omentin-1 (50 ng/mL) increased the accumulation of StAR protein in the endometrium on days 12 to 13 of pregnancy, and at the dose of 25 ng/mL also on days 10 to 12 of the oestrous cycle (Figure 2B,E).

Omentin-1 (50, 100 ng/mL) caused a decrease in the P450_SCC_ protein content on days 10 to 11 of gestation (Figure 2F), whereas at the doses of 25 and 100 ng/mL increased the abundance of the enzyme protein on days 10 to 12 of the oestrous cycle (Figure 2J).

Omentin-1 (50 ng/mL) increased 3βHSD protein expression on days 12 to 13 of pregnancy (Figure 2L), and at the doses of 25 and 100 ng/mL also during the oestrous cycle (Figure 2O). The adipokine at all doses reduced the enzyme protein abundance on days 15 to 16 of gestation (Figure 2M).

**Figure 3:**
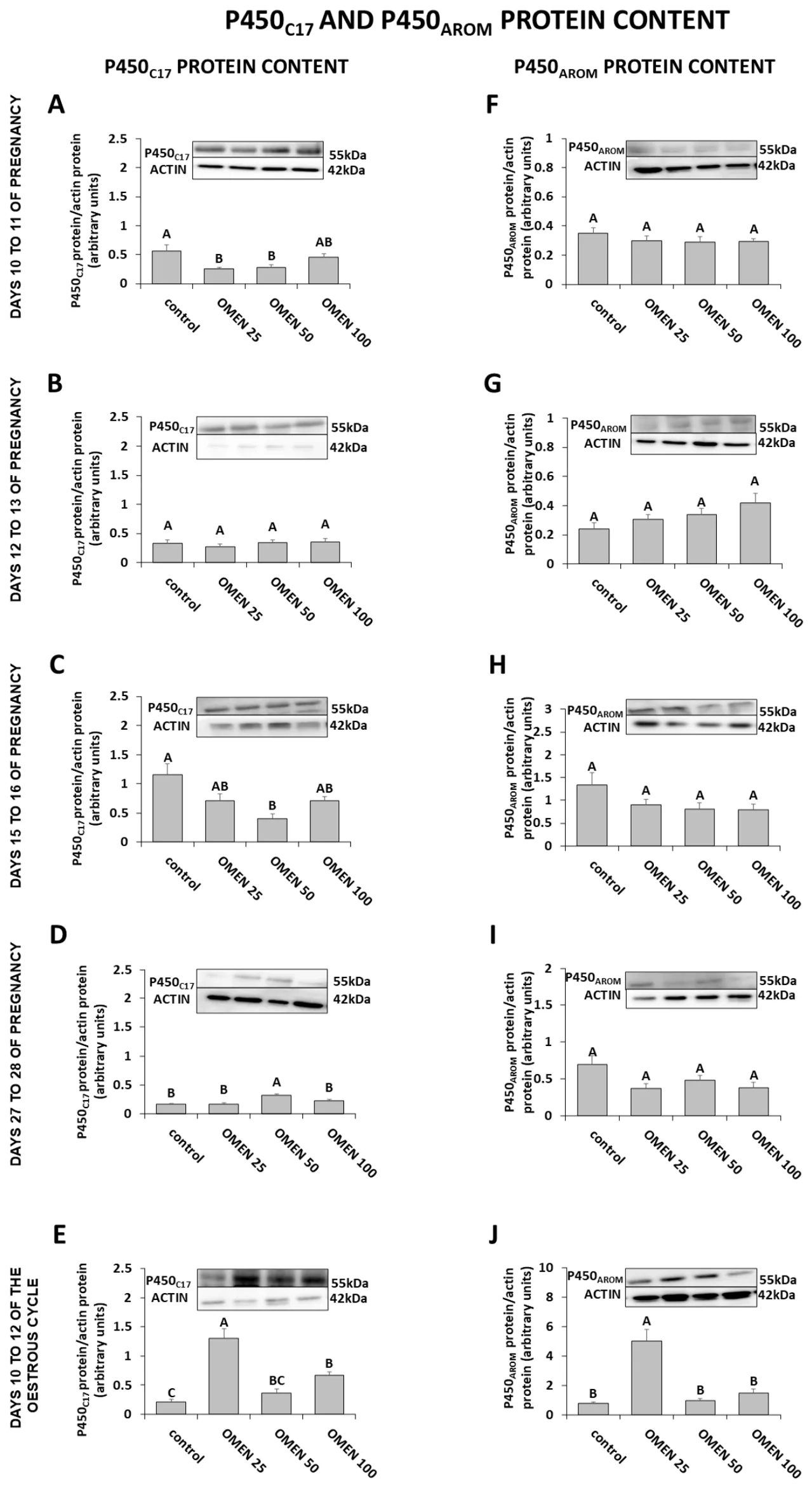
The effect of omentin-1 on the endometrial expression of cytochromes P450_C17_ and P450_AROM_ during the oestrous cycle and early pregnancy. The effect of omentin-1 (OMEN; 25, 50, and 100 ng/mL) on protein abundances of cytochrome P450_C17_ (P450_C17_; (**A**–**E**)) and cytochrome P450 aromatase (P450_AROM_; (**F**–**J**)) in the in vitro incubated endometrial explants obtained from pigs during early pregnancy (days 10 to 11, 12 to 13, 15 to 16, and 27 to 28) and on days 10 to 12 of the oestrous cycle. Upper panels: representative immunoblots; lower panels: protein content normalized to actin protein; results (arbitrary units) are presented as means ± S.E.M. (*n* = 5). Statistically significant differences are presented by different letters (*p* < 0.05).

Omentin-1 (50 ng/mL) increased P450_C17_ protein expression on days 27–28 of gestation (Figure 3D), and an increase was also observed during the oestrous cycle at doses of 25 and 100 ng/mL (Figure 3E). Adipokine decreased enzyme protein levels at doses of 25 and 50 ng/mL on days 10–11 of gestation (Figure 3A), and also at a dose of 50 ng/mL on days 15–16 (Figure 3C).

On days 10 to 12 of the oestrous cycle, the protein amounts of P450_AROM_ were elevated under the influence of omentin-1 at the dose of 25 ng/mL (Figure 3J).

### 2.3. The Effect of Omentin-1 on Akt Protein Phosphorylation in the Porcine Endometrium

**Figure 4:**
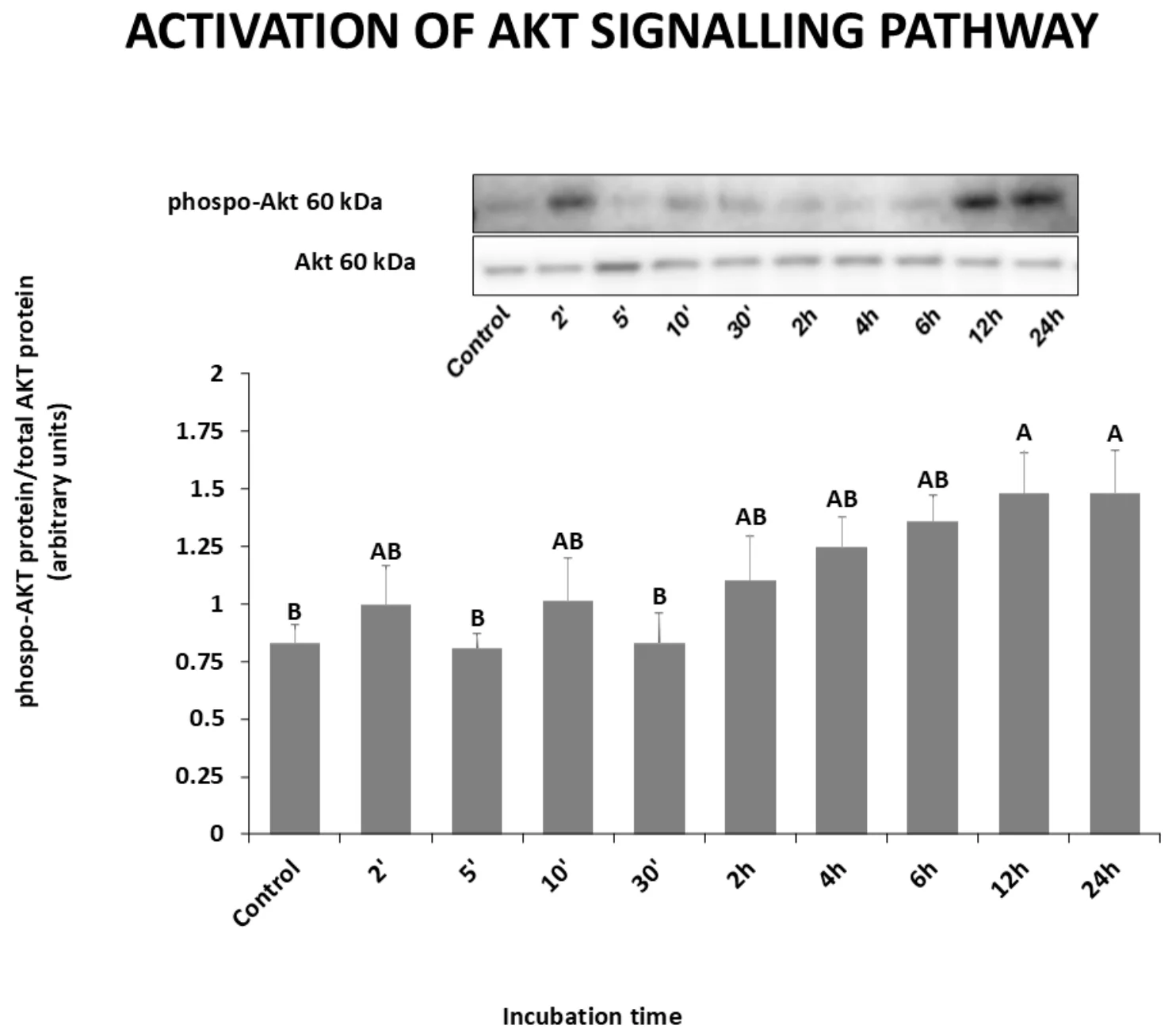
The effect of omentin-1 on the activation of the Akt signalling pathway in the endometrial tissue. The effect of omentin-1 (50 ng/mL) on phosphorylation of Akt protein in the in vitro incubated endometrial explants obtained from pigs on days 10 to 12 of the oestrous cycle. Upper panels, representative immunoblots; lower panels: phosphorylated protein content normalized to total protein content, results (arbitrary units) are presented as means ± S.E.M. (*n* = 5). Statistically significant differences are presented by different letters (*p* < 0.05).

Omentin-1 at the dose of 50 ng/mL significantly stimulated the phosphorylation of Akt protein after 12 and 24 h of incubation (*p* < 0.05; Figure 4).

### 2.4. The Effect of Omentin-1 on the Apoptosis of Endometrial LEc and STc

**Figure 5:**
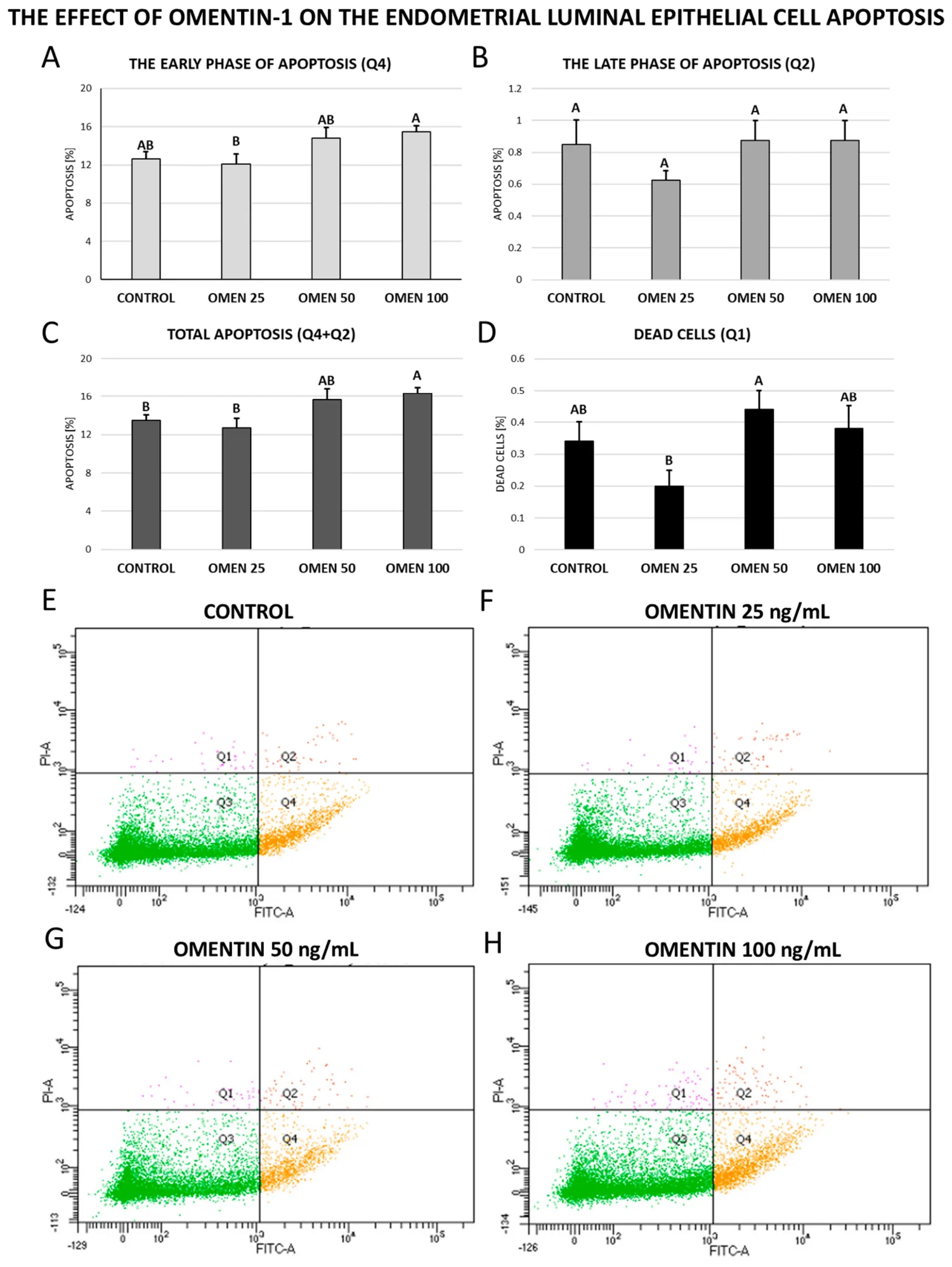
The in vitro effect of omentin-1 on the apoptosis process in LEc. The study was conducted on LEc obtained from pigs (*n* = 5) on days 15 to 16 of pregnancy. After isolation, cells were cultured till 90% confluency and then treated with omentin-1 (25, 50, or 100 ng/mL) or cultured in serum-free medium (control) for another 24 h. Following the completion of the in vitro culture, cell apoptosis was measured using flow cytometry and a commercially available kit for dual-color analysis (Annexin V and Propidium Iodide, PI). 20,000 events were used for both data collection and result analysis. A one-way analysis of variance (ANOVA) and Duncan’s post hoc test were used to assess the data. The findings are displayed as scatter plots (**E**–**H**) and mean ± S.E.M. (**A**–**D**). At *p* < 0.05, bars on graphs with distinct letters are deemed substantially different. The scatter plot’s quadrants, together with their corresponding numerical values, show the following: dead cells (Annexin V negative, PI positive) are shown in upper left (Q1), late apoptotic cells (Annexin V and PI positive) are shown in upper right (Q2), living cells (Annexin V and PI negative) are shown in lower left (Q3), and early apoptotic cells (Annexin V positive, PI negative) are shown in lower right (Q4).

Statistical analysis revealed that during the implantation onset, omentin-1 (100 ng/mL) increased the percentage of early apoptotic LEc, which contributed to the increased total percentage of apoptotic cells (Figure 5; *p* < 0.05). Omentin-1 did not affect the percentage of dead LEc (*p* > 0.05).

**Figure 6:**
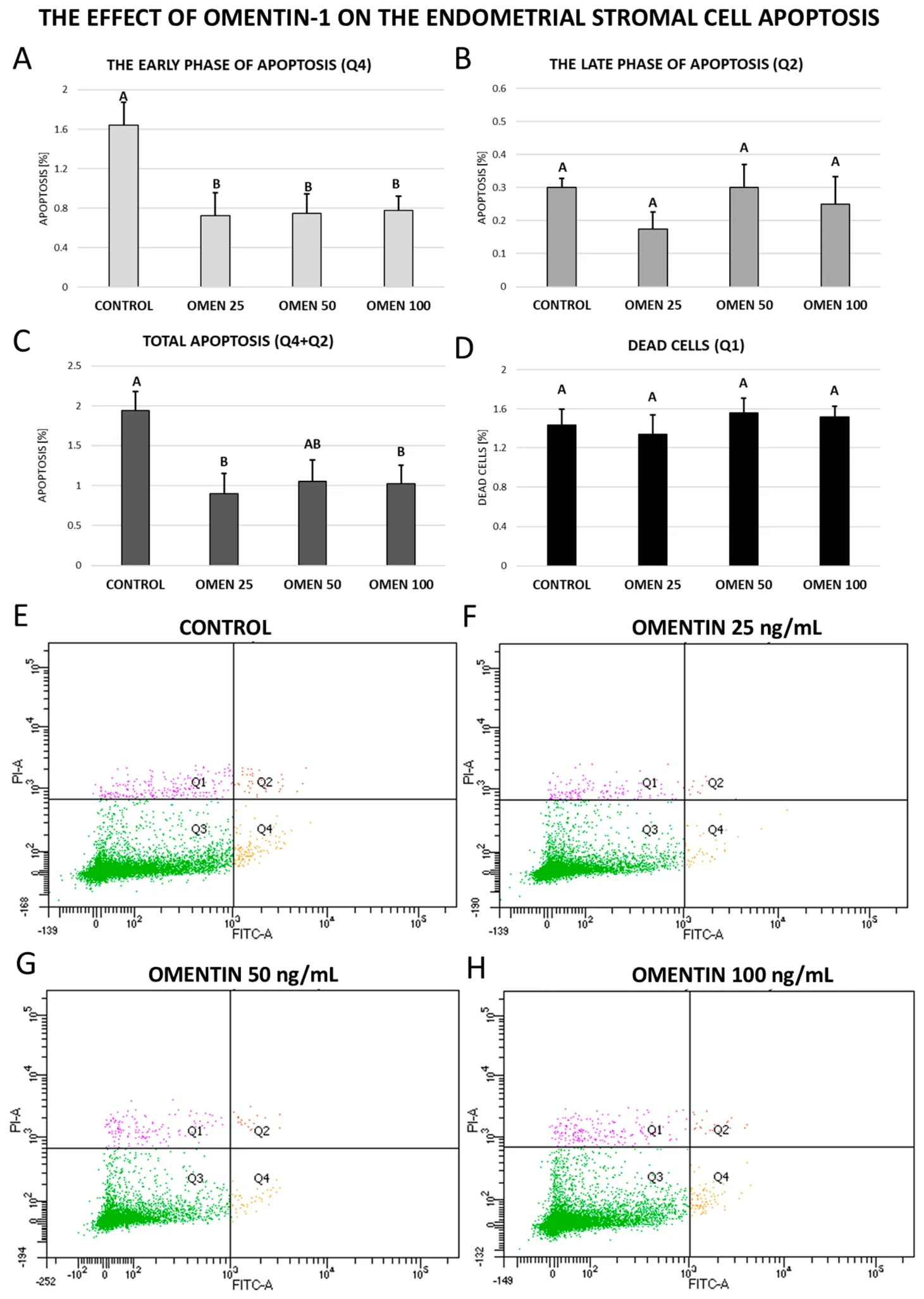
The in vitro effect of omentin-1 on the apoptosis process in STc. The study was conducted on STc obtained from pigs (*n* = 5) on days 15 to 16 of pregnancy. After isolation, cells were cultured till 90% confluency and then treated with omentin-1 (25, 50, or 100 ng/mL) or cultured in serum-free medium (control) for another 24 h. Following the completion of the in vitro culture, cell apoptosis was measured using flow cytometry and a commercially available kit for dual-color analysis (Annexin V and Propidium Iodide, PI). 20,000 events were used for both data collection and result analysis. A one-way analysis of variance (ANOVA) and Duncan’s post hoc test were used to assess the data. The findings are displayed as scatter plots (**E**–**H**) and mean ± S.E.M. (**A**–**D**). At *p* < 0.05, bars on graphs with distinct letters are deemed substantially different. The scatter plot’s quadrants, together with their corresponding numerical values, show the following: dead cells (Annexin V negative, PI positive) are shown in upper left (Q1), late apoptotic cells (Annexin V and PI positive) are shown in upper right (Q2), living cells (Annexin V and PI negative) are shown in lower left (Q3), and early apoptotic cells (Annexin V positive, PI negative) are shown in lower right (Q4).

Omentin-1 at all tested doses decreased the percentage of early apoptotic STc, which contributed to a decreased total percentage of apoptotic cells, at least for omentin-1 at the doses of 25 and 100 ng/mL (Figure 6; *p* < 0.05). No significant effect of omentin-1 was observed in the case of dead STc (*p* > 0.05).

### 2.5. The Effect of Omentin-1 on the Proliferation of Endometrial LEc and STc

**Figure 7:**
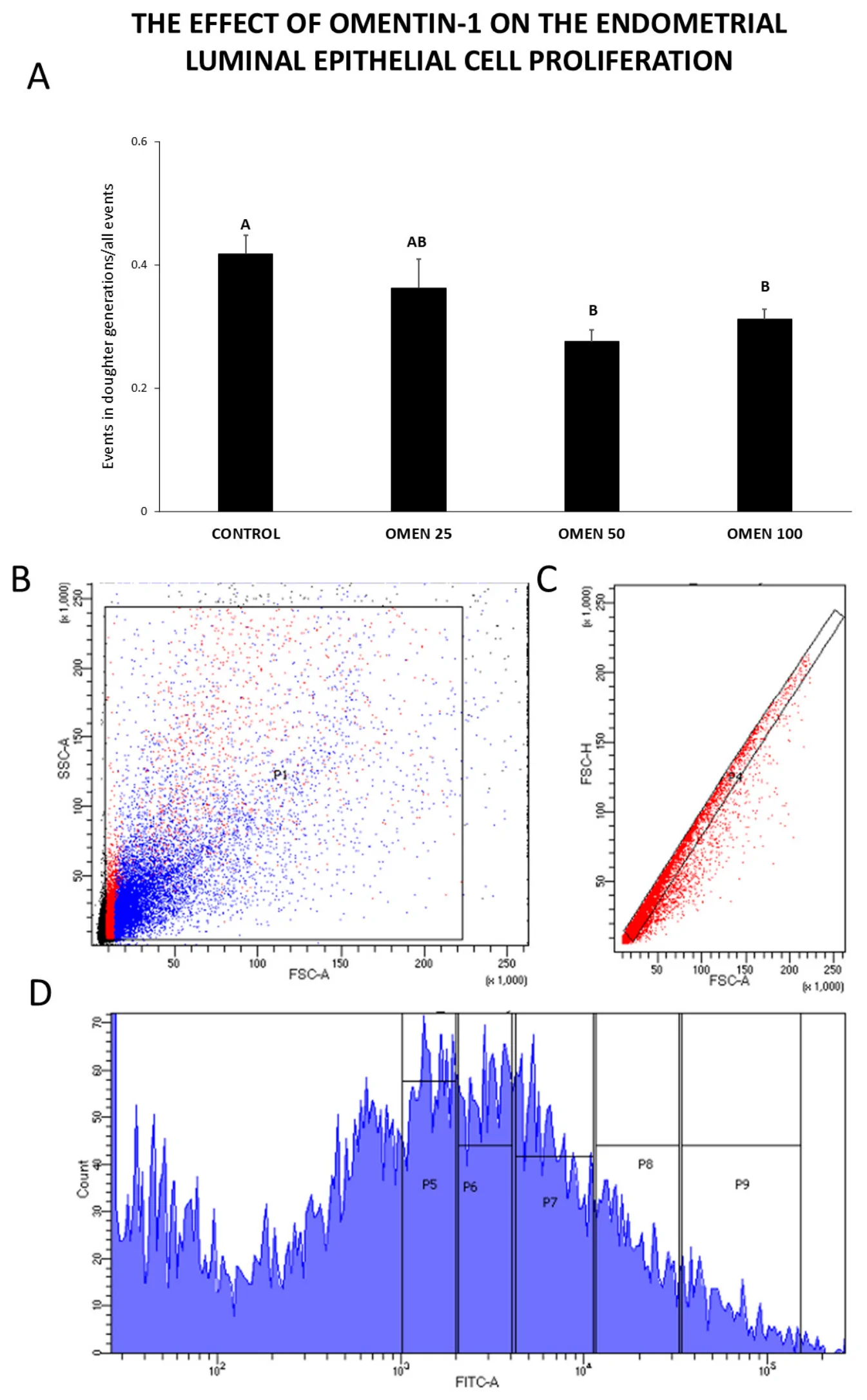
The in vitro effect of omentin-1 on the proliferation process in LEc. The study was conducted on LEc obtained from pigs (*n* = 5) on days 15 to 16 of pregnancy. After isolation, cells were cultured till 50% confluency. The cells were then stained with the CellTrace^TM^ proliferation kit and treated with omentin-1 (25, 50, or 100 ng/mL) or cultured in serum-free medium (control) for another 24 h. Next, LEc were analysed with flow cytometry. Data collection and analysis were carried out on 30,000 events. A one-way analysis of variance (ANOVA) and Duncan’s post hoc analysis were performed to assess the data. The results are presented as the mean proportion of cells in all daughter generations vs. all cells ± S.E.M. (**A**), representative flow cytometry dot plot showing side scatter area (SSC-A) versus forward scatter area (FSC-A) (**B**), doublet discrimination plot based on FSC-A versus FSC-H parameters (**C**), and histogram of FITC-A fluorescence intensity illustrating cell proliferation assessed by CellTrace^TM^ staining. The peaks within the defined gates represent successive generations of cells (**D**).

**Figure 8:**
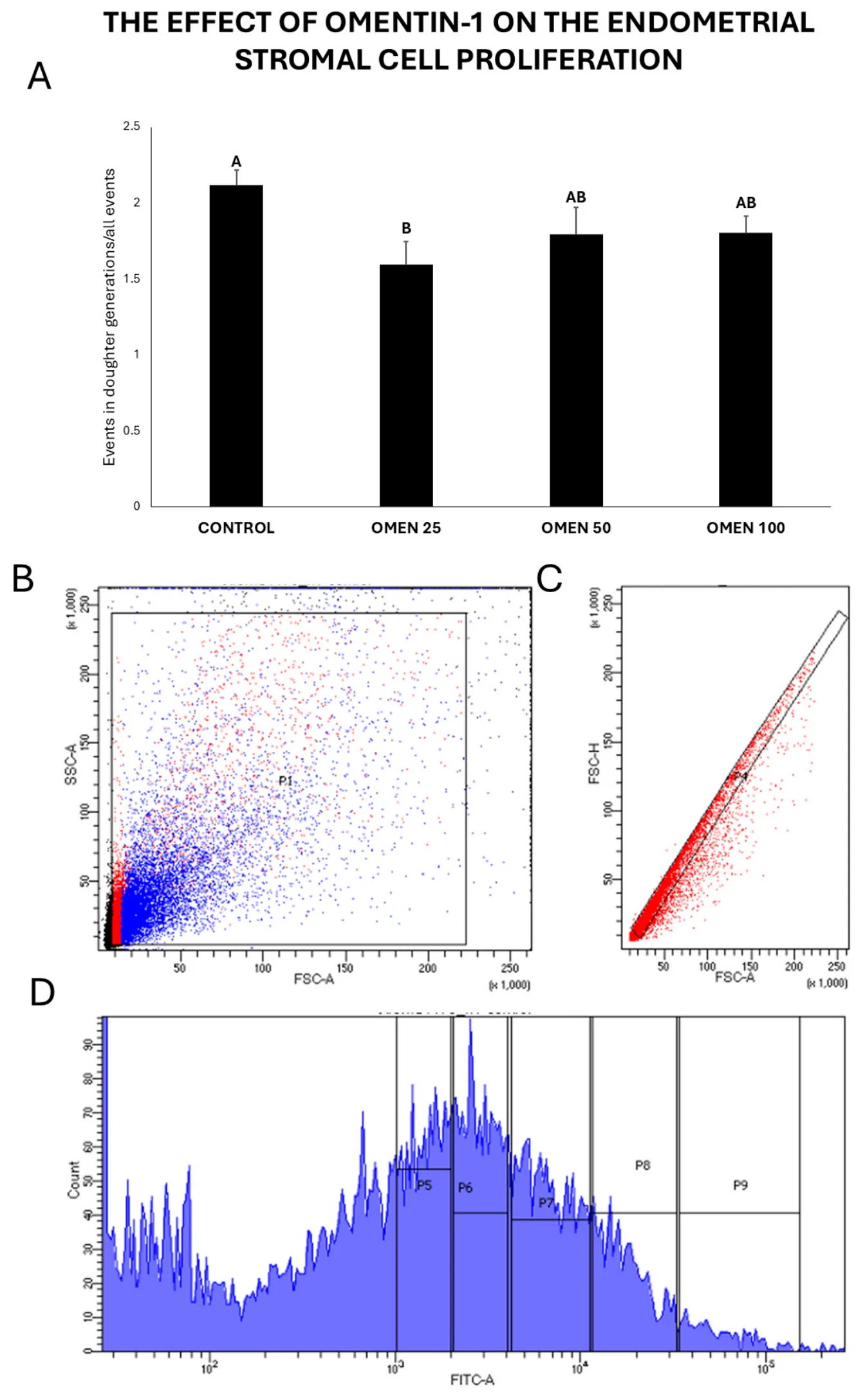
The in vitro effect of omentin-1 on the proliferation process in STc. The study was conducted on STc obtained from pigs (*n* = 5) on days 15 to 16 of pregnancy. After isolation, cells were cultured till 50% confluency. The cells were then stained with the CellTrace^TM^ proliferation kit and treated with omentin-1 (25, 50, or 100 ng/mL) or cultured in serum-free medium (control) for another 24 h. Next, STc were analysed with flow cytometry. Data collection and analysis were carried out on 30,000 events. A one-way analysis of variance (ANOVA) and Duncan’s post hoc analysis were performed to assess the data. The results are presented as the mean proportion of cells in all daughter generations vs. all cells ± S.E.M. (**A**), representative flow cytometry dot plot showing side scatter area (SSC-A) versus forward scatter area (FSC-A) (**B**), doublet discrimination plot based on FSC-A versus FSC-H parameters (**C**), and histogram of FITC-A fluorescence intensity illustrating cell proliferation assessed by CellTrace^TM^ staining. The peaks within the defined gates represent successive generations of cells (**D**).

Statistical analysis revealed that omentin-1 (50 and 100 ng/mL) decreased the proportion of dividing LEc compared to the control cells (Figure 7; *p* < 0.05). In the case of STc, omentin-1 (25 ng/mL) significantly decreased their proliferation potential (Figure 8; *p* < 0.05).

## 3. Discussion

To our knowledge, this is the first study demonstrating the in vitro effect of omentin-1 on steroidogenesis in the porcine endometrium during early pregnancy and the mid-luteal phase of the oestrous cycle, as well as its influence on tissue dynamics during implantation. Also, the involvement of omentin-1 in the activation of the Akt signalling pathway in endometrial cells was investigated. Collectively, our work provides evidence that omentin-1 acts as a metabolic–endocrine modulator of porcine endometrial function, supporting the coordination of steroidogenesis, intracellular signalling, and tissue remodelling in a stage-dependent manner. Omentin-1 appears to operate as a fine-tuning regulator, adjusting the local steroid environment and cellular dynamics in accordance with the physiological requirements of early pregnancy and the luteal phase. Although the porcine model does not directly reproduce human implantation, it provides a valuable large-animal system for studying local uterine mechanisms that are also relevant to human reproductive biology, including steroid-dependent endometrial receptivity, epithelial–stromal remodelling, metabolic signalling, and early pregnancy establishment [37]. In this context, omentin-1 may be considered not only as a regulator of porcine endometrial physiology, but also as a candidate metabolic signal linking energy balance with uterine competence [10].

The first major finding of the study is that omentin-1 promotes a P_4_-dominant local steroid environment, while modulating E_2_ secretion in a stage-dependent manner. This is physiologically important because the balance between P_4_ and oestrogens is central to endometrial receptivity, embryo–maternal communication, immune tolerance, and implantation-associated remodelling [3]. In humans, inadequate P_4_ responsiveness and disturbed endometrial steroid signalling are also considered important contributors to impaired receptivity and implantation failure [38]. Therefore, although the present data were obtained in pigs, they support the broader concept that adipokines may influence fertility by acting directly at the level of the uterus [39]. Omentin-1 increased both P_4_ and E_2_ secretion during embryo migration and the onset of placentation, whereas during maternal recognition of pregnancy and implantation onset, it shifted the local steroid environment toward P_4_ dominance. Such a shift during early pregnancy is essential for regulating implantation receptivity, immune tolerance, and prostaglandin signalling [40]. From a physiological perspective, such selective modulation is highly coherent. The present data suggest that omentin-1 may contribute to this process by biasing steroid flux toward P_4_, thereby supporting embryo survival.

The stage-dependent nature of omentin-1 action further strengthens its physiological relevance. During conceptus migration (days 10 to 11), the simultaneous increase in both P_4_ and E_2_ may reflect a requirement for transient uterine plasticity, where oestrogens support contractility and embryo distribution, while P_4_ maintains endometrial integrity [41]. Interestingly, omentin-1 increased P_4_ secretion at 100 ng/mL despite a reduction in P450_SCC_ protein abundance. Steroid concentrations measured in conditioned medium represent net steroid accumulation over the 24-h incubation period, whereas Western Blot provides an endpoint measure of protein abundance. Therefore, these two readouts do not necessarily change in parallel. However, the present study did not assess P450_SCC_ enzymatic activity, steroidogenic flux, substrate availability, or downstream steroid metabolism, and the mechanism underlying this apparent dissociation cannot be determined from the current data. Potential post-translational regulation, changes in substrate flux, or steroid turnover remain hypotheses that require direct experimental testing.

Maternal recognition of pregnancy in pigs is initiated around days 11 to 12, when the elongating conceptus produces a major oestrogen signal, and is subsequently supported by a temporally coordinated sequence of conceptus-derived signals, including IL1B and interferons, that modulate endometrial function through approximately days 15 to 16 of pregnancy [3]. In the present study, omentin-1 decreased E_2_ secretion by endometrial explants on days 12–13 while increasing P_4_ secretion under selected treatment conditions. Importantly, this effect concerns locally produced endometrial E_2_ and should be distinguished from the conceptus-derived oestrogen signal involved in maternal recognition of pregnancy. Thus, our results suggest that omentin-1 may contribute to the local adjustment of the endometrial steroid environment during this period rather than directly modulating the conceptus-derived pregnancy recognition signal. The increase in StAR protein during maternal recognition of pregnancy suggests enhanced mitochondrial cholesterol import, a rate-limiting step in P_4_ synthesis [42]. This mechanism is consistent with observations in ovarian cells, where omentin-1 enhances steroidogenesis via StAR and IGF-1-dependent pathways [30,34]. Moreover, simultaneously elevated 3βHSD abundance indicates that omentin-1 enhances P_4_ secretion through modulation of both substrate availability and enzyme abundance. Also consistent with context-dependent effects of omentin on ovarian steroidogenesis [34].

At the onset of conceptus attachment (days 15–16), omentin-1 decreased endometrial E_2_ secretion under selected treatment conditions and exerted cell-type-specific effects on isolated endometrial cells in vitro. Omentin-1 reduced LEc proliferation and increased apoptosis at the highest concentration tested, whereas in STc it reduced early apoptosis and affected proliferation only at selected concentrations. These observations demonstrate differential responsiveness of epithelial and stromal cells to omentin-1 under the present culture conditions. In pigs, conceptus attachment occurs at a non-invasive epitheliochorial interface in which the trophectoderm apposes an intact endometrial luminal epithelium, while the maternal–conceptus interface undergoes extensive structural reorganization and folding. Such remodelling is associated with coordinated changes in epithelial and stromal compartments and ultimately increases the surface area available for maternal–conceptus interactions [43,44]. Similar context-dependent effects of omentin-1 on proliferation and apoptosis have been reported in ovarian and mesenchymal models, where outcomes were dependent on cell type and signalling context [32,45,46]. The steroidogenic response observed at this stage provides an additional aspect of the omentin-1 effect. Simultaneous decrease in downstream enzymes (P450_C17_ or 3βHSD) indicates that omentin-1 may selectively restrict androgen and oestrogen pathways, thereby favouring a shift towards P_4_ in the uterine environment. Lower P450_C17_ may reduce excessive conversion of P_4_ toward androgen pathways. Taken together, the steroidogenic and cellular findings indicate that omentin-1 modifies several endometrial responses during the attachment period in vitro, while its contribution to physiological tissue remodelling and conceptus attachment in vivo remains to be established.

At the end of the implantation period (days 27–28), the simultaneous increase in both P_4_ and E_2_ under the influence of omentin-1 suggests a transition toward placentation and tissue expansion, where coordinated steroid signalling supports angiogenesis, immune modulation, and nutrient exchange [47]. In this phase, omentin-1 may facilitate the shift from implantation-specific remodelling to placental development, again acting in a context-dependent manner [48]. During this period, a steroidogenic profile like that observed during embryo migration was detected; however, unlike during conceptus migration, where both P450_C17_ and P450_SCC_ were reduced, we observed increased P450_C17_ abundance. Thus, it is physiologically justified that during transition toward placentation, the release of P_4_ is maintained stable and oestrogens are still available for angiogenesis/remodelling.

During the mid-luteal phase of the oestrous cycle, omentin-1 appears to exhibit a similar effect pattern to that seen during the same days of pregnancy. In this phase, E_2_ secretion is suppressed while P_4_ secretion levels increase. Unlike during the maternal recognition phase, in this stage, protein levels of all steroidogenic enzymes are elevated. This pattern is consistent with a fine-tuning role for omentin-1 in maintaining a uterine environment compatible with luteal dominance. Such modulation may enhance endometrial steroidogenic capacity, supporting readiness for possible fertilization, or may participate in preparing the endometrium for subsequent luteal-phase outcomes depending on the presence or absence of conceptus-derived signals. Additionally, suppression of E_2_ despite enhanced P450_AROM_ during the mid-luteal phase of the oestrous cycle suggests post-translational or indirect regulation through broader changes in steroid metabolism, rather than that the adipokine directly regulates aromatase-dependent oestrogen synthesis. This situation may also reflect oestrogen conversion pathways to fine-tune sensitivity, including increased conversion of E_2_ to the less active E_1_ via 17βHSD activity [5,49,50].

Mechanistically, the omentin-1-induced increase in Akt phosphorylation provides a potential link between metabolic signalling and reproductive function. This observation is consistent with its known insulin-sensitizing properties and its ability to modulate cell survival and steroidogenesis [51]. Previous studies on ovarian cells have shown that omentin-1 can activate insulin-related signalling cascades, including PI3K/Akt, thereby influencing proliferation and metabolic activity [34]. In the endometrium, Akt signalling has been implicated in the regulation of cell proliferation and implantation-associated cellular responses, while studies in steroidogenic reproductive cells demonstrate that PI3K/Akt can also modulate steroid hormone production [52,53]. Thus, Akt may represent a signalling node linking metabolic and cellular responses in reproductive tissues, although its role in coordinating endometrial steroidogenesis remains to be established.

Another key aspect is that omentin-1 likely acts within a broader network of adipokines rather than regulating alone [19,20,21]. Increasing evidence indicates that adipokines collectively regulate reproductive tissues by integrating metabolic status with local endocrine and inflammatory responses [34]. Therefore, the effects observed here should be interpreted as part of a multi-adipokine regulatory system, where omentin-1 contributes to fine-tuning rather than overriding existing pathways.

The present findings may also have broader relevance for human reproductive disorders associated with metabolic imbalance [54]. Omentin-1 is closely linked with insulin sensitivity, adipose tissue function, obesity-related metabolic disturbance, and inflammatory status [55]. These factors are also clinically relevant in conditions such as obesity-associated infertility, Polycystic Ovary Syndrome (PCOS), endometriosis, and recurrent implantation failure, where altered steroid responsiveness, impaired endometrial receptivity, chronic low-grade inflammation, and disturbed epithelial–stromal communication may compromise fertility. Omentin-1’s role in influencing steroid secretion, Akt phosphorylation, cell growth, and apoptosis in the endometrium, therefore, supports the hypothesis that metabolic adipokines may play a direct role in preparing the uterus for implantation [51]. Nevertheless, this interpretation remains speculative and requires validation in human endometrial cells, organoids, or tissue explants before clinical relevance can be established [56].

Several aspects of the present study warrant further investigation. Endometrial explants comprise multiple cell populations, including epithelial, stromal, endothelial, immune, and vascular-associated cells; therefore, this model does not allow the specific cellular sources of steroid production or the cell types responding to omentin-1 to be identified. Future studies employing purified cell populations, spatial approaches, or single-cell analyses could help resolve these cell-specific responses. Transcript-level analyses could provide complementary information on the transcriptional regulation of steroidogenic genes, although mRNA and protein abundance reflect distinct levels of regulation and do not necessarily change in parallel at a given time point [57]. Accordingly, future studies combining transcript and protein measurements with direct assessment of steroidogenic enzyme activity or steroid flux would help distinguish transcriptional, post-transcriptional, and functional regulation of endometrial steroidogenesis by omentin-1. Finally, although omentin-1 increased Akt phosphorylation, the present study did not include pharmacological inhibition or genetic perturbation of candidate signalling components. Pathway-specific inhibitors and knockdown approaches will therefore be valuable for establishing the causal involvement of Akt and other candidate signalling pathways in omentin-1-mediated endometrial responses.

## 4. Materials and Methods

### 4.1. Experimental Animals

The experiments were conducted according to the Polish Act of the protection of animals used for educational or scientific purposes of 15 January 2015 (Polish Journal of Law of 2015, item 266; available online: https://dziennikustaw.gov.pl/DU/2015/266; accessed on 14 January 2022) and Directive 2010/63/EU of the European Parliament of 22 September 2010 on the protection of animals used for scientific objectives. All tissues used in the study were abattoir by-products; therefore, no ethical committee approval was necessary.

25 female pigs (7–8 months of age, 130–140 kg) were fed according to current Polish standards, using a balanced diet (balanced protein level, the addition of exogenous amino acids, minerals, micro- and macro-elements). Animals had access to freshwater ad libitum. For the assessment of the effects of omentin-1 on endometrial steroidogenesis, experimental grouping of female pigs was performed according to the phase of the pregnancy or oestrous cycle (*n* = 5 per group) as follows: days 10 to 11 (the transuterine migration of embryos), 12 to 13 (the maternal recognition of pregnancy), 15 to 16 (the embryo attachment and initiation of implantation), 27 to 28 (the end of implantation), and days 10 to 12 of the oestrous cycle (the mid-luteal phase; the peak activity of corpora lutea, similar to that seen during the beginning of gestation). For the evaluation of the effect of omentin-1 on the proliferation and apoptosis of luminal (LEc) and stromal cells (STc), only tissues from days 15 to 16 of pregnancy were used. The day of the onset of the second oestrus was considered as day 0 of the oestrus cycle. Gilts were inseminated naturally on days 1 to 2 of the cycle. After slaughter, uteri were transported to the laboratory on ice-cold in phosphate-buffered saline (PBS) enriched with 1% Antibiotic–Antimycotic solution (Sigma Aldrich, USA). Next, the uterine horns were washed three times with PBS containing antibiotics to remove conceptuses, trophoblast, and uterine fluid. Based on ovarian morphology, the stage of the oestrous cycle was confirmed [58]. The day of gestation was assessed by the presence and morphology of embryos collected from the uterine horns [59].

### 4.2. Tissue Collection and In Vitro Cultures of Endometrial Explants

In vitro cultures of endometrial explants were performed according to [22] in five independent experiments (*n* = 5 animals per group) for each studied phase. Endometrial explants dissected from the uterine horns (100 mg ± 10%) were washed three times in M199 medium (Sigma Aldrich, St. Louis, MO, USA). Individual endometrial slices were placed in separate glass vials with 2 mL of M199 medium containing 0.1% Bovine Serum Albumin (BSA) (TargetMol, Boston, MA, USA), 5% foetal calf serum (Sigma-Aldrich, USA), and 1% Antibiotic–Antimycotic Solution (Sigma-Aldrich, USA). Tissue cultures were preincubated in a shaking water bath for 2 h at 37 °C in 95% humidified air containing 5% CO_2_. For the evaluation of omentin-1 effects on the protein expression of steroidogenic enzymes and steroid secretion, after preincubation, slices were treated with omentin-1 (25, 50, and 100 ng/mL; R&D Systems, Minneapolis, MN, USA) and incubated for an additional 24 h or cultured without any treatment (control samples) under the same conditions. For the evaluation of the adipokine effects on the activation of the Akt signalling pathway, the explants were treated with omentin-1 at a concentration of 50 ng/mL for 0, 2, 5, 10, and 30 min, as well as for 2, 4, 6, 12, and 24 h. The omentin-1 concentrations used in the in vitro experiments were chosen based on physiological concentrations reported in the porcine blood plasma [36] and our preliminary measurements of the adipokine concentrations in the porcine plasma and uterine luminal fluid (ULF), 41–140 ng/mL and 37–64 ng/mL, respectively (Kiezun et al., unpublished data). Following incubation, media and explants were collected and stored at −20 and −80 °C, respectively. The viability of tissue explants was determined by measuring lactate dehydrogenase (LDH) activity using the Liquick Cor-LDH kit (Cormay, Lomianki, Poland) according to the manufacturer’s instructions. The activity of LDH in the media during the culture of the endometrial slices was compared with its activity in homogenates after the tissue homogenization (positive control of cell death and the maximal release of LDH). The mean activities of LDH in media after culture were as follows: control samples—6.22 ± 1.87 U/L (0.66% of maximal release of LDH), omentin-1 at the dose of 25 ng/mL—32.88 ± 6.4 U/L (3.61% of maximal release of LDH), omentin-1 at the dose of 50 ng/mL—51.8 ± 6.03 U/L (5.53% of maximal release of LDH), omentin-1 at the dose of 100 ng/mL—23.33 ± 1.28 U/L (2.49% of maximal release of LDH).

### 4.3. Radioimmunoassay (RIA)

Radioimmunoassay (RIA) was used to measure the quantities of P_4_ and E_2_ in medium [21,22]. Dziadkowiec et al. [56] and Szafranska et al. [57] have documented previously the cross-reactivities of antisera against investigated steroids.

The no-extraction assay was used to assess P_4_ levels. For the E_2_ measurement, the extraction efficiency was 90.67 ± 0.73%. The test had sensitivities of 0.5 pg/mL for E_2_ and 1 pg/mL for P_4_. P_4_ standard curve ranged from 1 to 1500 pg/mL, while E_2_ ranged from 0.5 to 200 pg/mL. For P_4_, the intra- and inter-assay coefficients of variation were 1.86 ± 0.33% and 8.37%, while for E_2_, they were 1.06 ± 0.39% and 7.74%.

### 4.4. Protein Isolation and Western Blot

The T-PER^TM^ Mammalian Protein Extraction Reagent (Thermo Fisher Scientific, Waltham, MA, USA) with Halt Protease and Phosphatase Inhibitor Single-Use Cocktail was used to homogenize endometrial tissue samples on ice. Lysates were centrifuged twice (4 °C, 10 min, 10,000× *g*) after being incubated on ice for 30 min. The samples were subsequently frozen at −80 °C for further examination. The Bradford method was utilized to measure protein levels using the Infinite M200 Pro (Tecan, Männedorf, Switzerland). With few adjustments, Western blot analysis was carried out in the manner described by Gudelska et al. [19]. Sodium dodecyl sulfate polyacrylamide gel electrophoresis (SDS-PAGE) was used to separate the same volume of total lysates (20 µg) on 12.5% polyacrylamide gels. Semi-dry electroblotting onto 0.45 μm PVDF membranes (Whatman-Amersham, Amersham, UK) was carried out following electrophoresis. The membranes were then blocked for 1.5 h at room temperature (RT) in Tris-buffered saline that contained 0.1% Tween 20 and 5% skimmed milk powder. Following that, rabbit polyclonal primary antibodies against StAR, P450_SCC_, 3βHSD, P450_C17_, P450_AROM_, and actin, which served as a reference protein, were applied to the membranes overnight. The membranes were washed in TBST and then incubated with goat anti-rabbit secondary antibodies coupled with horseradish peroxidase (HRP) for 1.5 h. When the molecular masses were sufficiently different, the PVDF membrane was cut horizontally after transfer, and the corresponding sections of the same membrane were incubated separately with antibodies against the target protein and actin. In cases when the molecular masses of the target protein and actin were close, the same membrane was sequentially probed, stripped (Restore™ Western Blot Stripping Buffer, Thermo Fisher Scientific, USA), and reprobed to actin protein. In the experiments in which the molecular masses were indistinguishable, the target protein and loading control were detected on matched parallel membranes loaded with the same sample aliquots in the same order with the same amount of protein. Full uncropped representative blots are presented in Supplementary File S1. Antibodies, as well as other details of the Western Blot method used in the study, were specified in Table 1. Nonspecific foetal calf serum (instead of primary antibodies) was used to generate negative control blots. Further, isotype controls were performed with the use of Rabbit IgG control antibodies instead of primary antibodies for target proteins. No unspecific bands were observed in negative control blots. Negative control as well as isotype control blots are presented in Supplementary File S2. Immunocomplexes were visualized using chemiluminescence (Immobilon, Merck Millipore, Burlington, MA, USA). Membranes were analysed and archived using Azzure (Azzure Biosystems, Dublin, CA, USA) with chemifluorescence and chemiluminescence imaging software (Intuitive Capture Software, Azzure Biosystems). Concentrations of the studied proteins were quantified by densitometric analysis of immunoblots using Image Studio Lite v. 5.2.

### 4.5. Isolation and Culture of Porcine Endometrial LEc and STc

The analysis of omentin-1 effects on the processes of apoptosis and proliferation was performed on isolated endometrial LEc and STc. Porcine uterine horns were collected after embryo flushing and immediately rinsed twice with sterile phosphate-buffered saline (PBS) containing antibiotics to remove residual material. Each uterine horn was filled with 60 mL of enzymatic solution containing 0.5% dispase (Merck, Rahway, NJ, USA) and 0.25% pancreatin (Merck, USA) prepared in HBSS buffer, sealed with surgical clamps, and externally decontaminated by immersion for 5 min in sterile PBS supplemented with NaClO (1 mL/L). The horns were subsequently transferred to fresh sterile PBS and subjected to enzymatic digestion in a shaking water bath at 37 °C for 45 min. After digestion, the HBSS solution containing detached LEc was collected, and the uterine horns were additionally rinsed five times with 40 mL of fresh HBSS to recover remaining cells. The pooled cell suspension was filtered through sterile 250 µm gauze filters to remove tissue fragments and large cell aggregates and centrifuged (227× *g*, 10 min, RT). The cell pellet was resuspended in red blood cell lysis buffer (Merck, USA) and incubated for 5 min at RT in the dark. The suspension was then diluted with warm M199 medium (Merck, USA) supplemented with 0.1% BSA (Merck, USA) and centrifuged again under the same conditions. Washing in M199 medium containing 0.1% BSA was repeated three times. Following the final wash, cells were resuspended in M199 medium supplemented with 10% foetal calf serum (FCS; Merck, USA) and 1% BSA and transferred to 25-mL culture flasks for 6 h pre-incubation at 37 °C in a humidified atmosphere of 95% air and 5% CO_2_. This differential adhesion step allowed removal of contaminating STc and fibroblasts, which attach more rapidly to plastic surfaces than LEc. After pre-incubation, the non-adherent cell fraction enriched in LEc was collected, centrifuged (227× *g*, 10 min), and resuspended in fresh culture medium. Cell number and viability were determined using trypan blue exclusion (Merck, USA) and a Bürker hemocytometer (Fuchs-Rosenthal, Lauda-Königshofen, Germany). Cells were seeded onto collagen-coated 6-well culture plates (Collagen Coating Solution, Merck, USA) at a density of 2 × 10^6^ cells per well in 2 mL of M199 medium supplemented with 10% FCS and 1% BSA and cultured at 37 °C in a humidified incubator (5% CO_2_). Culture medium was replenished or replaced every 48 h.

In the case of endometrial STc, the endometrial stromal tissue was dissected from the uterine horns. The tissue was cut into small pieces and digested for 45 min in 0.2% collagenase (Merck, USA) solution in HBSS (37 °C). Cell suspension was filtered through sterile 75 µm cell filters to remove tissue fragments and large cell aggregates, and centrifuged (227× *g*, 10 min, RT). The cell pellet was resuspended in red blood cell lysis buffer and incubated for 5 min at RT in the dark. The suspension was then diluted with warm M199 medium supplemented with 0.1% BSA and centrifuged again under the same conditions. Washing in M199 medium containing 0.1% BSA was repeated three times. Following the final wash, cells were resuspended in M199 medium supplemented with 10% foetal calf serum and 1% BSA. The number of cells and their viability were evaluated exactly as described for LEc. Cells were then seeded on TC-treated 6-well plates at a density of 2 × 10^6^ cells per well in 2 mL of M199 medium supplemented with 10% FCS and 1% BSA and cultured at 37 °C in a humidified incubator (5% CO_2_). Culture medium was replenished or replaced every 48 h.

### 4.6. Cell Apoptosis Assessment with Flow Cytometry Technique

For both types of cells, upon reaching approximately 90% confluence, the culture medium was removed, and cells were incubated for 24 h in serum- and BSA-free M199 medium (control) or in medium supplemented with omentin-1 (25, 50, and 100 ng/mL). After treatment, cells were detached using 0.05% trypsin and 0.02% EDTA (Merck, USA), collected by centrifugation (227× *g*, 10 min, RT), and the resulting cell pellets were used for the evaluation of apoptosis.

The apoptosis of LEc and STc was determined by dual-color flow cytometry analysis. The procedure was examined according to the manufacturer’s instructions for the Swine Annexin V-Fluorescein Apoptosis Assay Kit (ImmunoChemistry Technologies, Davis, CA, USA). Cells in the early phase of apoptosis were labeled by the green-fluorescent Annexin V, while cells in the late stage of apoptosis, as well as dead cells, were labeled by the red-fluorescent Propidium Iodide (PI) stain. As a positive control for apoptosis, cells were treated with camptothecin (Merck, USA) at 4 µg/mL, following the recommendations provided in the Apoptosis Assay Kit protocol. At the end of the in vitro culture period, the medium was removed, and the cells were dissociated with 0.05% trypsin–EDTA (Merck, USA). After double rinsing with PBS, the cells were centrifuged at 227× *g* for 5 min at RT. Next, the cells were suspended at a concentration of 1 × 10^6^ in 0.4 mL of a calcium-based binding buffer. FACSCelesta™ flow cytometer (BD Biosciences, Franklin Lakes, NJ, USA) was used for flow cytometric measurements. Data were acquired and analysed using FACSDiva 9.0 software (BD Biosciences, USA). Both data acquisition and outcome analysis were performed on 20,000 events.

### 4.7. Cell Proliferation Assessment with Flow Cytometry Technique

The analysis of proliferation of LEc and STc was determined with CellTrace^TM^ CFSE Cell Proliferation Kit (Thermo Fisher Scientific, Waltham, MA, USA) according to the manufacturer’s protocol. The method is based on the covalent binding of the cell-permeant fluorescent dye carboxyfluorescein succinimidyl ester (CFSE) to intracellular proteins. During successive cell divisions, the fluorescent signal is equally partitioned between daughter cells, resulting in a progressive halving of fluorescence intensity that can be quantified by flow cytometry.

For both types of cells, upon reaching approximately 50% confluence, the culture medium was removed, and cells were stained with CellTrace^TM^ CFSE diluted 1:1000 in sterile PBS for 20 min, protected from light, and placed in the culture conditions. Next, the staining solution was removed, and fresh M199 medium containing 1% BSA was added for 5 min (37 °C). Afterwards, the medium was removed, and cells were incubated for another 72 h in serum- and BSA-free M199 medium (control) or in medium supplemented with omentin-1 (25, 50, and 100 ng/mL). At the end of the in vitro culture period, the medium was removed, and the cells were dissociated with 0.05% trypsin–EDTA (Merck, USA). After double rinsing with PBS, the cells were centrifuged at 227× *g* for 5 min at RT. Next, the cell pellet was resuspended in PBS and analysed with the FACSCelesta™ flow cytometer (BD Biosciences, USA). Data were acquired and analysed using the FACSDiva 9.0 software (BD Biosciences, USA). The number of cells in each generation was quantified and expressed as the proportion of divided cells (sum of events in all daughter generations/sum of events in all generations, including the parent generation), which was calculated as an overall measure of proliferative activity. Both data acquisition and outcome analysis were performed on 30,000 events.

### 4.8. Statistical Analysis

All the data obtained were assessed with the Shapiro–Wilk test for the assumption of normality and Levene’s test for homogeneity of variance with the use of Statistica v. 13 software (StatSoft, Tulsa, OK, USA). The results were examined using one-way ANOVA and Duncan’s post hoc test to ascertain the effects of omentin-1 at various concentrations on protein expression and hormone secretion by the endometrial tissue explants, as well as to assess the impact of omentin-1 on the proliferation and apoptosis of LEc and STc. Data are expressed as mean values ± S.E.M. based on five independent experiments (*n* = 5 per group). Differences were considered statistically significant at *p* < 0.05.

## 5. Conclusions

**Figure 9:**
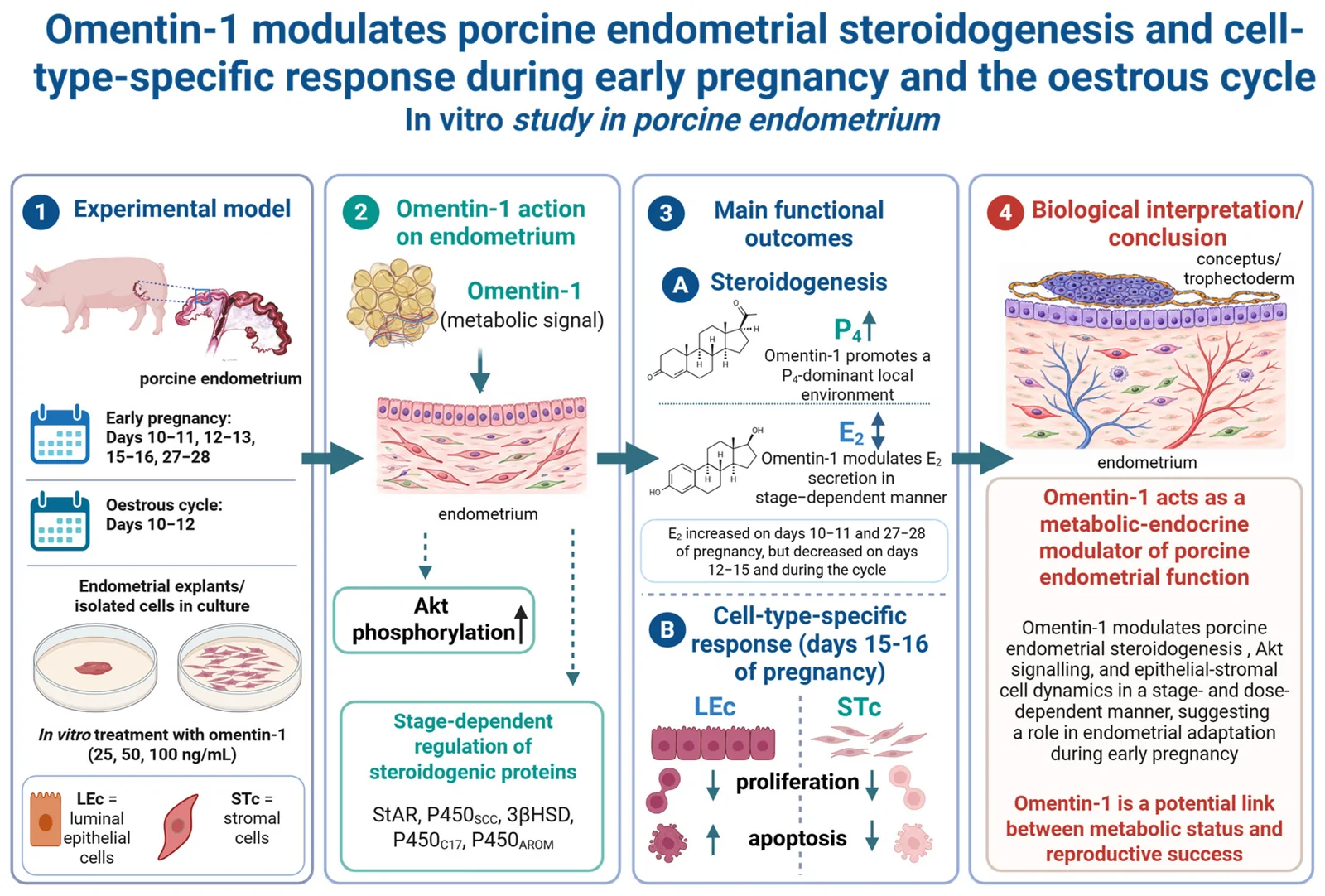
Graphical summary of the effects of omentin-1 on porcine endometrial function during early pregnancy and the oestrous cycle. Porcine endometrial explants and isolated luminal epithelial cells (LEc) and stromal cells (STc) were treated in vitro with omentin-1. Omentin-1 promoted a progesterone-dominant local steroid environment, modulated oestradiol secretion and the abundance of steroidogenic proteins in a stage-dependent manner, and stimulated Akt phosphorylation. During conceptus attachment, omentin-1 decreased the proliferation of both LEc and STc, increased apoptosis in LEc, and reduced apoptosis in STc. Collectively, these findings indicate that omentin-1 acts as a metabolic–endocrine modulator of porcine endometrial steroidogenesis, intracellular signalling, and epithelial–stromal remodelling. Created in BioRender. Kiezun, M. (2026) https://BioRender.com/7cmtar5 (accessed on 26 August 2026).

Taken together, the data support a model in which omentin-1 acts as a metabolic–endocrine modulator of porcine endometrial function, adjusting steroidogenesis and tissue remodelling according to the stage of early pregnancy or the luteal phase of the oestrous cycle. Its actions are characterised by promotion of progesterone-dominant conditions, selective modulation of steroidogenic pathways, activation of Akt signalling, and cell-type-specific regulation of proliferation and apoptosis. This combination of effects positions omentin-1 as a previously unrecognised regulator of endometrial adaptation and suggests that adipokines may represent an important mechanistic link between metabolic status and reproductive success. Although further studies are required to determine whether similar mechanisms operate in the human endometrium, the present findings provide a useful framework for future research on metabolic regulation of uterine receptivity in obesity-, PCOS-, endometriosis-, and infertility-related contexts. Thus, omentin-1 may be one component of the adipokine-mediated dialogue through which metabolic status is translated into local uterine responses required for successful reproduction (Figure 9).

## Figures and Tables

**Table 1:** **Characteristics of the antibodies used in the study.**

Protein	Host Species	Catalogue Number and Supplier	Primary Antibody Dilution	Blocking Agent	Secondary Antibodies	Secondary Antibody Dilution
StAR	Rabbit	ab96637 (Abcam, Cambridge, UK)	1:250	5% milk in TBST	Goat anti-rabbit (AP156P; Merck Millipore, USA)	1:2000
P450_SCC_		ab175408 (Abcam, UK)	1:250			1:2000
P450_C17_		ab125022 (Abcam, UK)	1:250			1:2000
P450_AROM_		A2161 (ABclonal, Woburn, MA, USA)	1:250			1:2000
3βHSD		SAB2101086 (Sigma Aldrich, St. Louis, MO, USA)	1:200			1:2000
Actin		A2066 (Sigma Aldrich, USA)	1:2000			1:20,000
Akt		#9272 (Cell Signaling Technology, Boston, MA, USA)	1:1000			1:20,000
phospho-Akt		#9271 (Cell Signaling Technology, USA)	1:500			1:20,000

StAR, steroidogenic acute regulatory protein; P450_SCC_, P450 side-chain cleavage enzyme; P450_C17_, cytochrome P450 C17; P450_AROM_, cytochrome P450 aromatase; 3βHSD, 3β-hydroxysteroid dehydrogenase; Akt, protein kinase B.

## Data Availability

The original contributions presented in this study are included in the article/supplementary material. Further inquiries can be directed to the corresponding author.

## Supplementary Materials

The following supporting information can be downloaded at: https://www.mdpi.com/article/10.3390/ijms27177731/s1.

## Abbreviations

The following abbreviations are used in this manuscript:

P4	Progesterone
E2	Oestradiol
E1	Oestrone
A	Androstenedione
T	Testosterone
INSR	Insulin receptor
IGF-1	Insulin-like Growth Factor 1
ERK1/2	Extracellular signal-related kinases 1 and 2
PKA	Protein Kinase A
Akt	Protein Kinase B
OMEN	Omentin-1
StAR	Steroidogenic acute regulatory protein
P450_SCC_	P450 side-chain cleavage enzyme
3βHSD	3β-hydroxysteroid dehydrogenase
P450_C17_	Cytochrome P450_C17_
P450_AROM_	Cytochrome P450 aromatase
LEc	Luminal cells
STc	Stromal cells
PGF2α	Prostaglandin F2 alpha
PBS	Phosphate-buffered saline
17βHSD	17β-Hydroxysteroid dehydrogenase
PI3K	Phosphoinositide 3-kinase
PCOS	Polycystic Ovary Syndrome
BSA	Bovine Serum Albumin
ULF	Uterine luminal fluid
LDH	Lactate dehydrogenase
RIA	Radioimmunoassay
SDS-PAGE	Sodium dodecyl sulphate polyacrylamide gel electrophoresis
RT	Room temperature
HRP	Horseradish peroxidase
FCS	Foetal calf serum
PI	Propidium Iodide
CFSE	Carboxyfluorescein succinimidyl ester
