# Supplementary material for: Omentin-1 Modulates Porcine Endometrial Steroidogenesis and Tissue Remodelling During Early Pregnancy and the Oestrous Cycle
Source: Int J Mol Sci. 2026 Aug 28;27(17):7731. doi: 10.3390/ijms27177731 (PMC13565993; doi:10.3390/ijms27177731)

Negative control blots

1. Negative control blots were performer by the use of nonspecific foetal calf serum diluted in TBST instead of the primary antibody. Besides, the whole Western Blot procedure was performed exactly as described in the Materials and Methods section. Random protein samples were used for negative blot preparation.

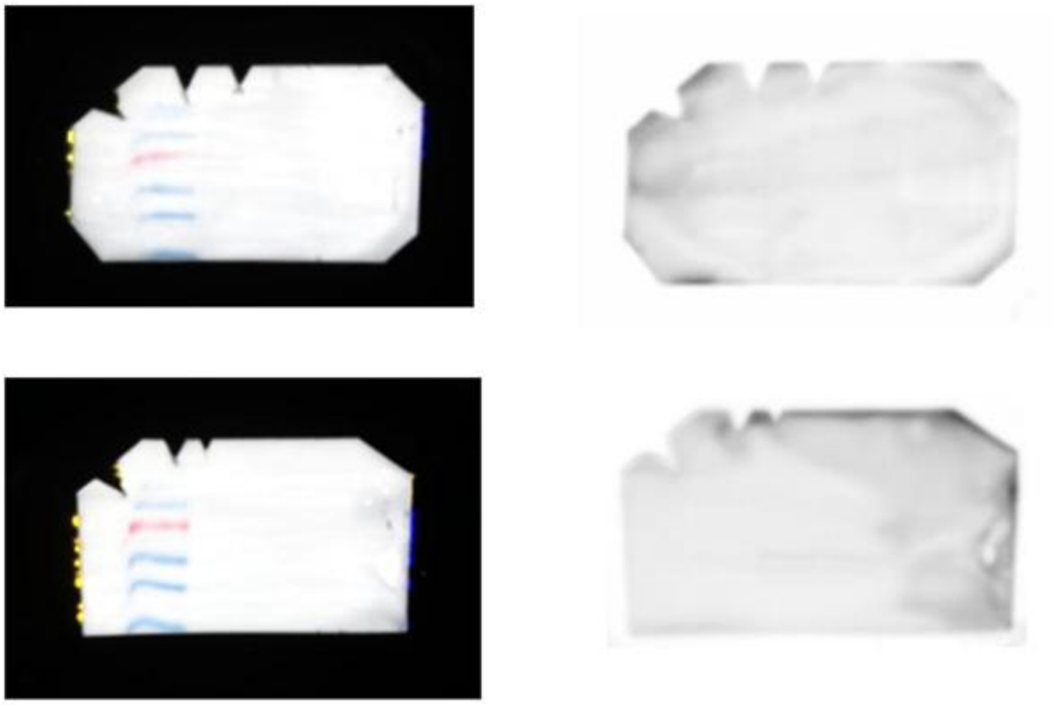

2. Additionally, we performed isotype controls of primary antibodies used in the study. Isotype controls were performed with the use of Rabbit IgG control antibodies instead of primary antibodies for target proteins. Besides, the whole Western Blot procedure was performed exactly as described in the Materials and Methods section. Random protein samples were used for isotype control blot preparation.

a) StAR

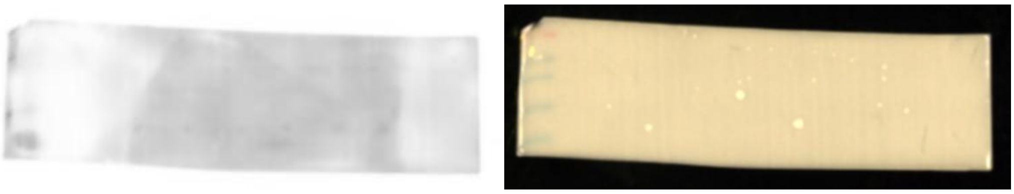

b) P450SCC

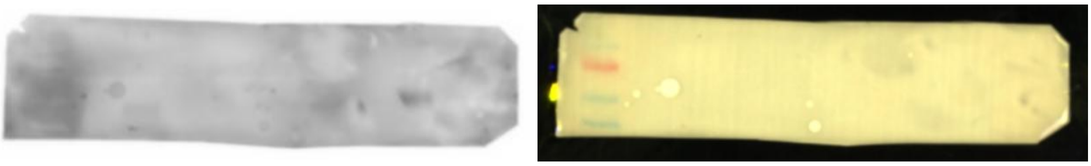

c) 3βHSD

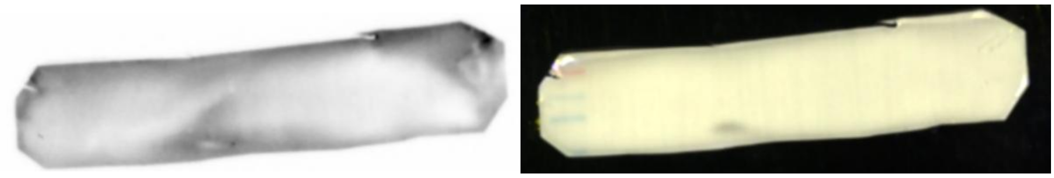

d) P450C17

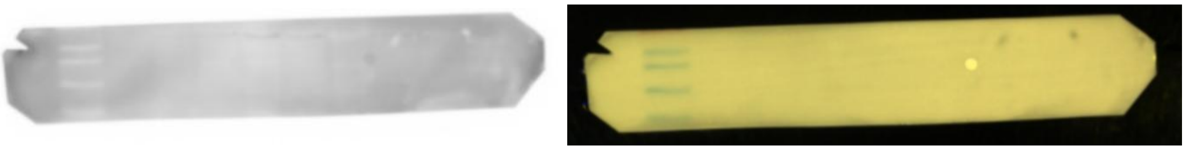

e) P450AROM

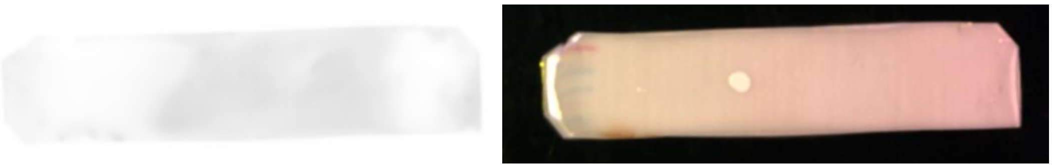

Supplement: Supplementary file 1 [file ijms-27-07731-s001.zip › Supplementary File S2 negative controls.pdf]
