# Supplementary material for: Omentin-1 Modulates Porcine Endometrial Steroidogenesis and Tissue Remodelling During Early Pregnancy and the Oestrous Cycle
Source: Int J Mol Sci. 2026 Aug 28;27(17):7731. doi: 10.3390/ijms27177731 (PMC13565993; doi:10.3390/ijms27177731)
Supplement: Supplementary file 1 [file ijms-27-07731-s001.zip › Supplementary Tables S1-S5.pdf]

Table S1. The one-way ANOVA analysis's results. Effects of the main factor on the progesterone (P4) and oestradiol (E2) production by the porcine endometrium.

			PROGESTERONE		ESTRADIOL	
			F	*p*	F	*P*
DAYS OF PREGNANCY	10 to 11	OMENTIN	3.70117	0.042821	15.747	0.000049
	12 to 13	OMENTIN	6.74048	0.006453	4.170	0.023225
	15 to 16	OMENTIN	4.16787	0.030776	3.722	0.033318
	27 to 28	OMENTIN	4.02325	0.034031	6.6043	0.004112
DAYS 10 to 12 OF THE OESTROUS CYCLE		OMENTIN	4.78698	0.020358	10.908	0.000380

Table S2. The one-way ANOVA analysis's results. Effects of the main factor on the steroidogenic acute regulatory protein (StAR), P450 side-chain cleavage enzyme (P450SCC), 3-hydroxysteroid dehydrogenase (3βHSD), cytochrome P450C17 (P450C17), cytochrome P450 aromatase (P450AROM) abundances by the porcine endometrium.

			STAR		P450 SCC		3BHSD		P450 C17		P450 AROM	
			F	*p*	F	*p*	F	*p*	F	*p*	F	*p*
DAYS OF PREGNANCY	10 to 11	OMENTIN	2.2864	0.117754	4.1600	0.023406	1.4488	0.265836	3.6256	0.036067	0.5938	0.628051
	12 to 13	OMENTIN	8.61925	0.001236	3.9267	0.028194	8.2655	0.001508	0.3929	0.759821	2.0447	0.148132
	15 to 16	OMENTIN	1.9362	0.164451	0.2282	0.875380	4.7868	0.014466	4.6417	0.016132	1.8032	0.187159
	27 to 28	OMENTIN	0.1103	0.952821	2.5501	0.092192	1.5635	0.237085	7.9453	0.001812	2.4908	0.097355
DAYS 10 to 12 OF THE OESTROUS CYCLE		OMENTIN	17.3865	0.000027	23.6868	0.000004	24.5073	0.000003	18.5182	0.000019	17.90403	0.000023

Table S3. The one-way ANOVA analysis's results. Effects of the main factor on the phosphorylation of Akt protein by the porcine endometrium.

Akt	F	*P*
INCUBATION TIME WITH OMENTIN	2.5066	0.022271

Table S4. The one-way ANOVA analysis's results. Effects of the main factor on the apoptosis of LEc and STc in the porcine endometrium.

		THE EARLY PHASE OF APOPTOSIS		THE LATE PHASE OF APOPTOSIS		TOTAL APOPTOSIS		DEAD CELLS	
		F	*p*	F	*p*	F	*p*	F	*P*
LEc	OMENTIN	3.1034	0.056219	1.3394	0.296703	3.7765	0.031853	2.6478	0.084327
STc	OMENTIN	3.85232	0.029944	0.91184	0.457277	3.13099	0.054886	0.3035	0.822444

Table S5. The one-way ANOVA analysis's results. Effects of the main factor on the prof of LEc and STc in the porcine endometrium.

Events in doughter generations/all events		F	*p*
OMENTIN	LEc	4.0686	0.025164
	STc	2.4059	0.105319
