# Supplementary material for: Omentin-1 Modulates Porcine Endometrial Steroidogenesis and Tissue Remodelling During Early Pregnancy and the Oestrous Cycle
Source: Int J Mol Sci. 2026 Aug 28;27(17):7731. doi: 10.3390/ijms27177731 (PMC13565993; doi:10.3390/ijms27177731)
Supplement: Supplementary file 1 [file ijms-27-07731-s001.zip › Supplementary File S1 uncropped blots.pdf]

Figure 2. (A) The protein expression level of StAR in endometrial explants was detected by Western blot (n = 5).

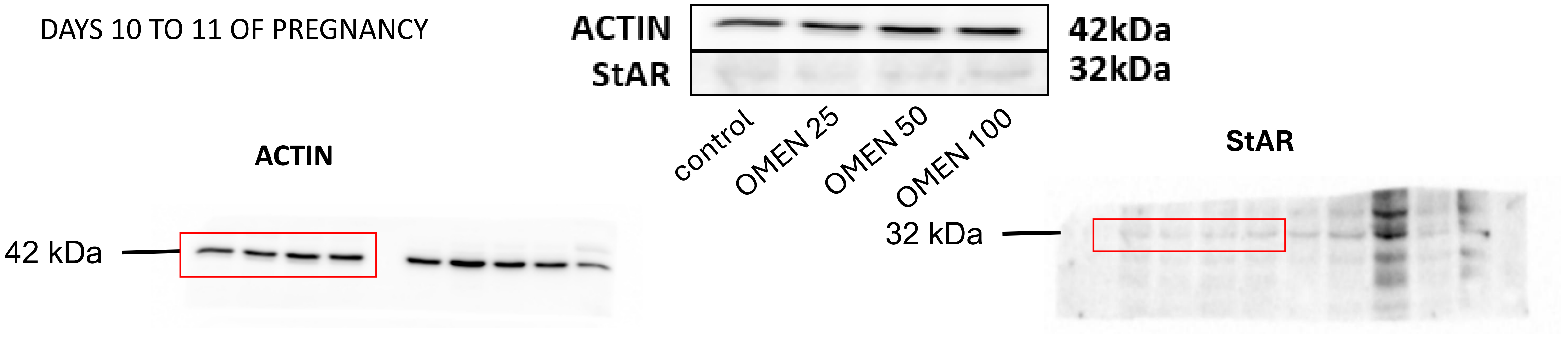

Molecular marker: Thermo Scientific™ PageRuler™
Prestained Protein Ladder, 10 to 180 kDa, 11812124

Figure 2. (B) The protein expression level of StAR in endometrial explants was detected by Western blot (n = 5).

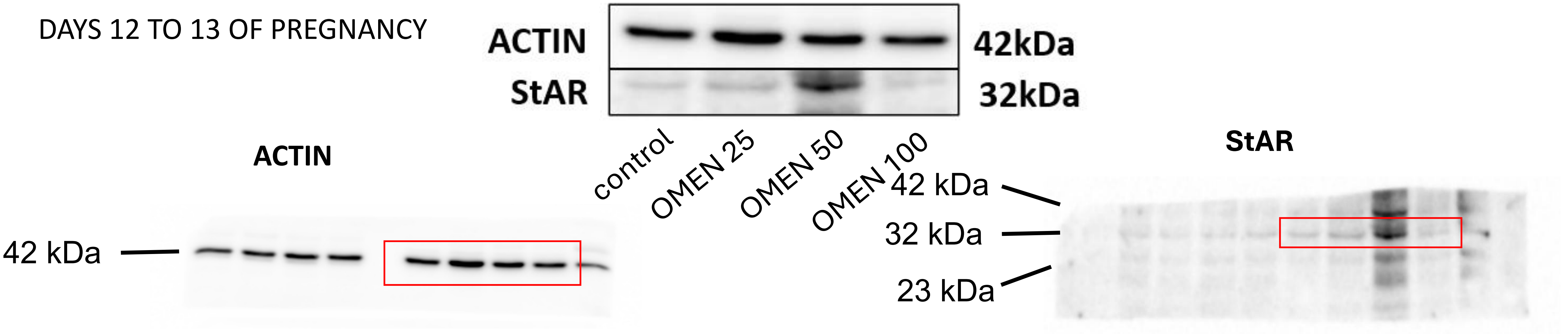

Molecular marker: Thermo Scientific™ PageRuler™
Prestained Protein Ladder, 10 to 180 kDa, 11812124

Figure 2. (C) The protein expression level of StAR in endometrial explants was detected by Western blot (n = 5).

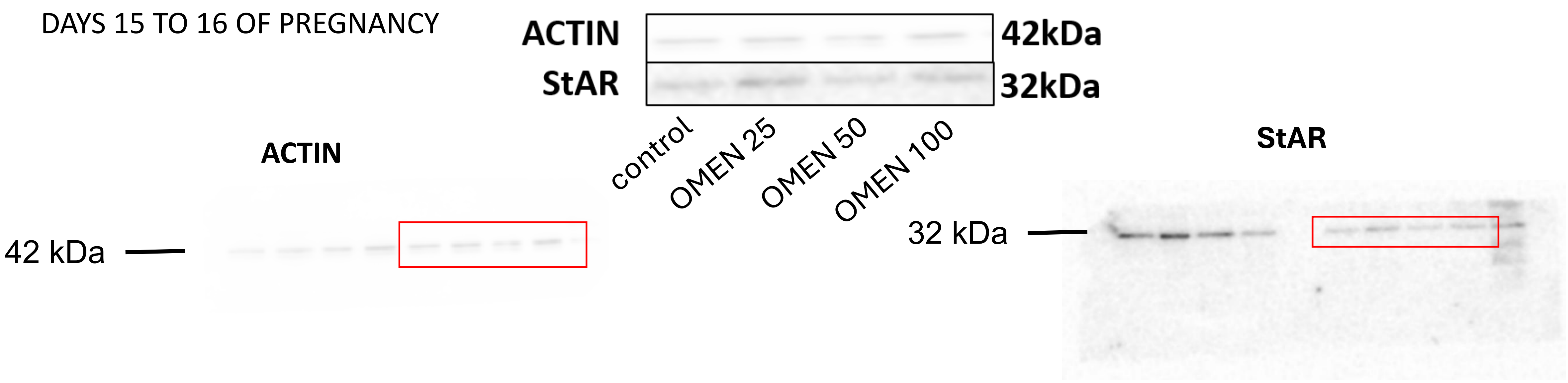

Molecular marker: Thermo Scientific™ PageRuler™
Prestained Protein Ladder, 10 to 180 kDa, 11812124

Figure 2. (D) The protein expression level of StAR in endometrial explants was detected by Western blot (n = 5).

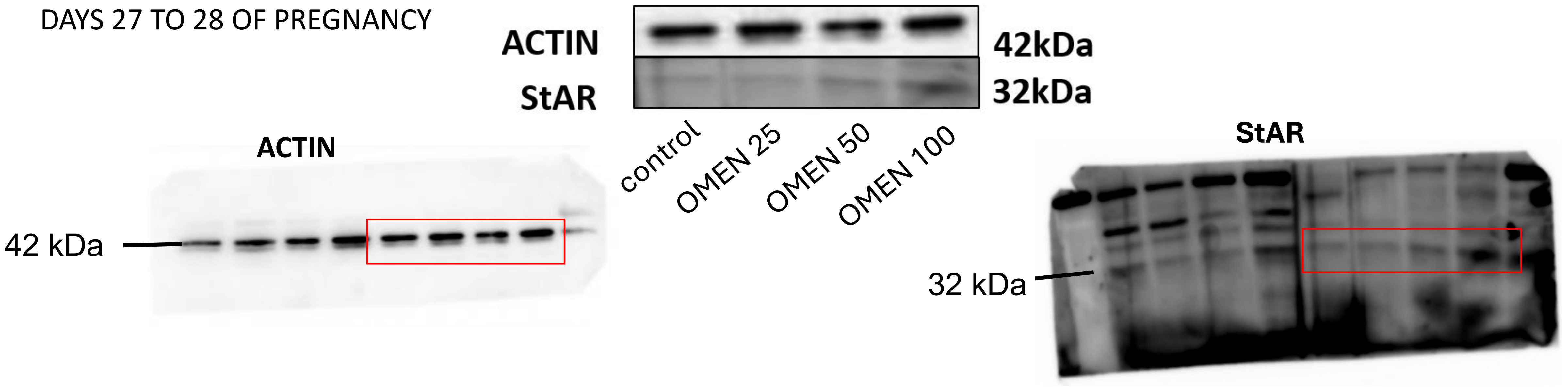

Molecular marker: Thermo Scientific™ PageRuler™
Prestained Protein Ladder, 10 to 180 kDa, 11812124

Figure 2. (E) The protein expression level of StAR in endometrial explants was detected by Western blot (n = 5).

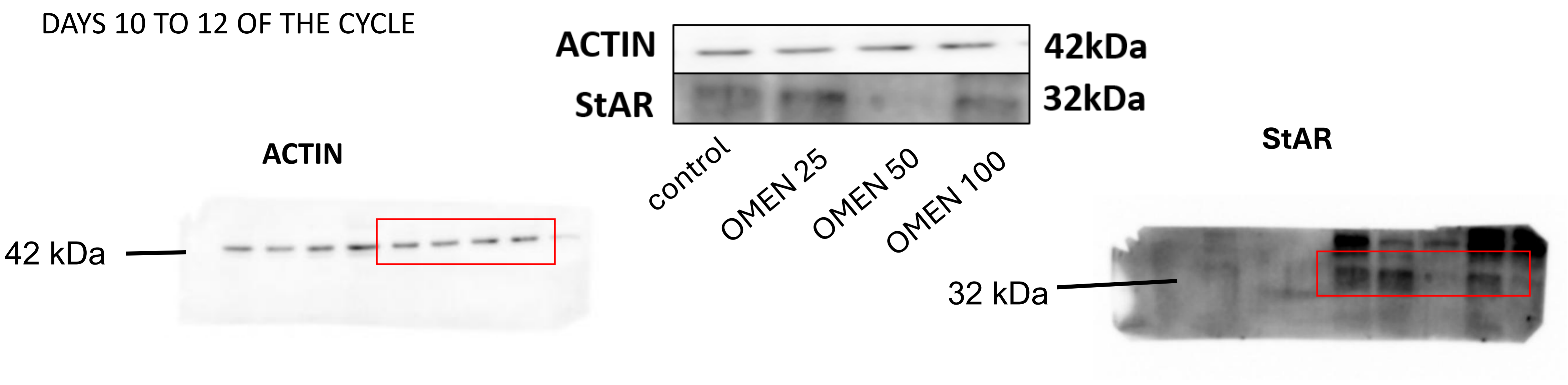

Molecular marker: Thermo Scientific™ PageRuler™
Prestained Protein Ladder, 10 to 180 kDa, 11812124

Figure 2. (F) The protein expression level of $P450_{SCC}$ in endometrial explants was detected by Western blot (n = 5).

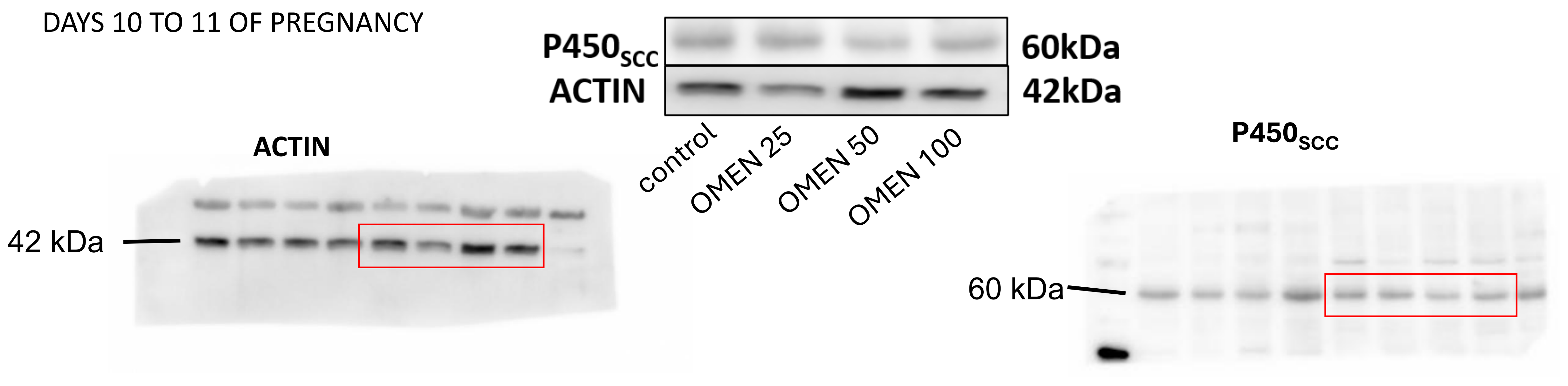

Molecular marker: Thermo Scientific™ PageRuler™
Prestained Protein Ladder, 10 to 180 kDa, 11812124

Figure 2. (G) The protein expression level of $P450_{SCC}$ in endometrial explants was detected by Western blot (n = 5).

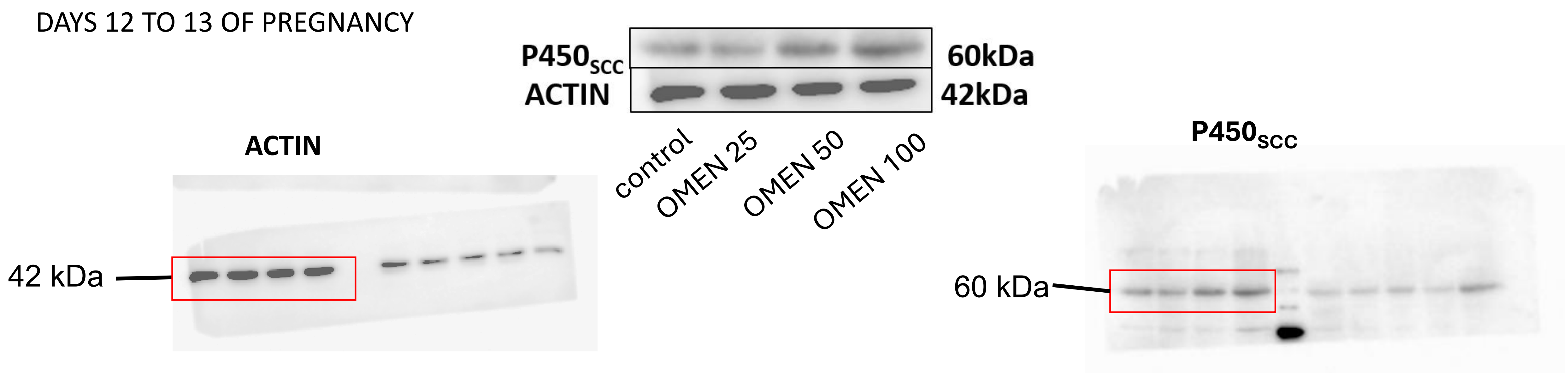

Molecular marker: Thermo Scientific™ PageRuler™
Prestained Protein Ladder, 10 to 180 kDa, 11812124

Figure 2. (H) The protein expression level of $P450_{SCC}$ in endometrial explants was detected by Western blot (n = 5).

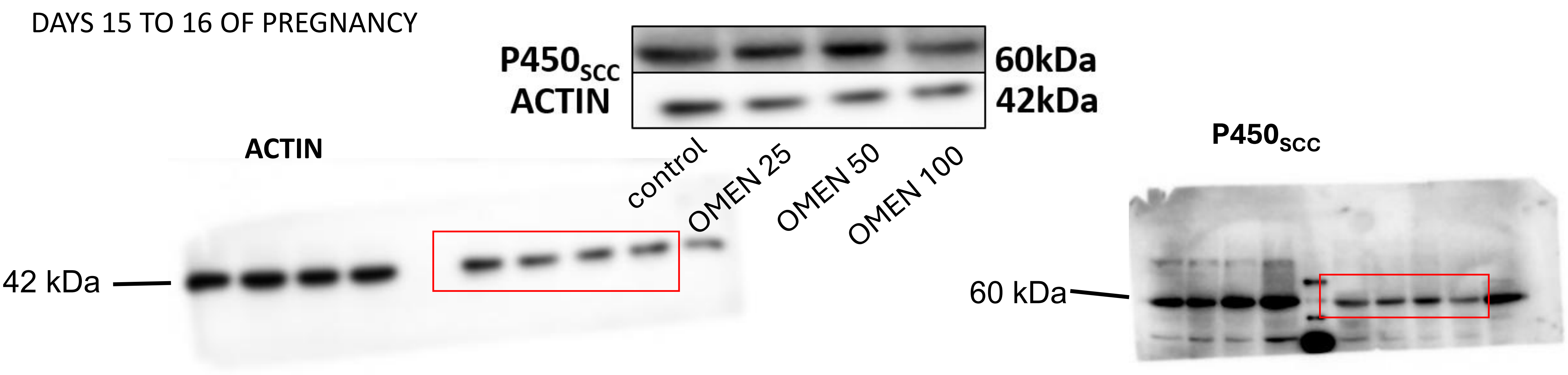

Molecular marker: Thermo Scientific™ PageRuler™
Prestained Protein Ladder, 10 to 180 kDa, 11812124

Figure 2. (I) The protein expression level of $P450_{SCC}$ in endometrial explants was detected by Western blot (n = 5).

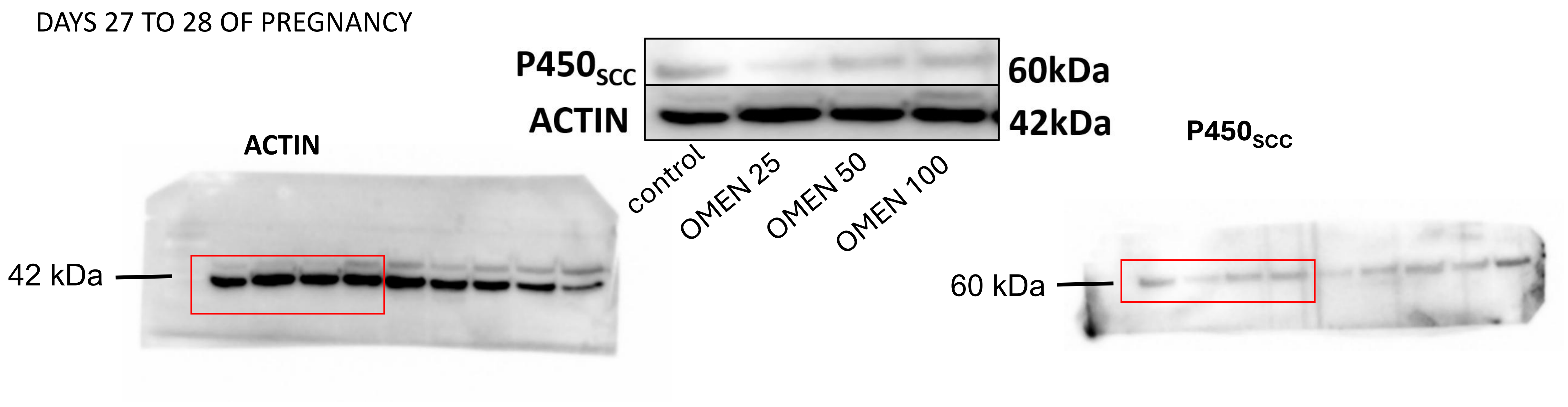

Molecular marker: Thermo Scientific™ PageRuler™
Prestained Protein Ladder, 10 to 180 kDa, 11812124

Figure 2. (J) The protein expression level of $P450_{SCC}$ in endometrial explants was detected by Western blot (n = 5).

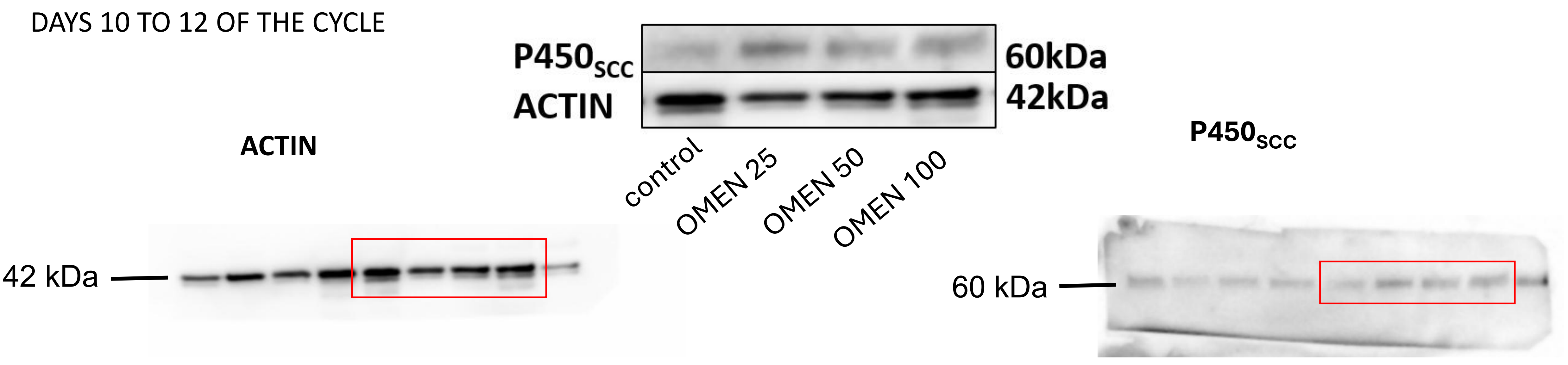

Molecular marker: Thermo Scientific™ PageRuler™
Prestained Protein Ladder, 10 to 180 kDa, 11812124

Figure 2. (K) The protein expression level of 3βHSD in endometrial explants was detected by Western blot (n = 5).

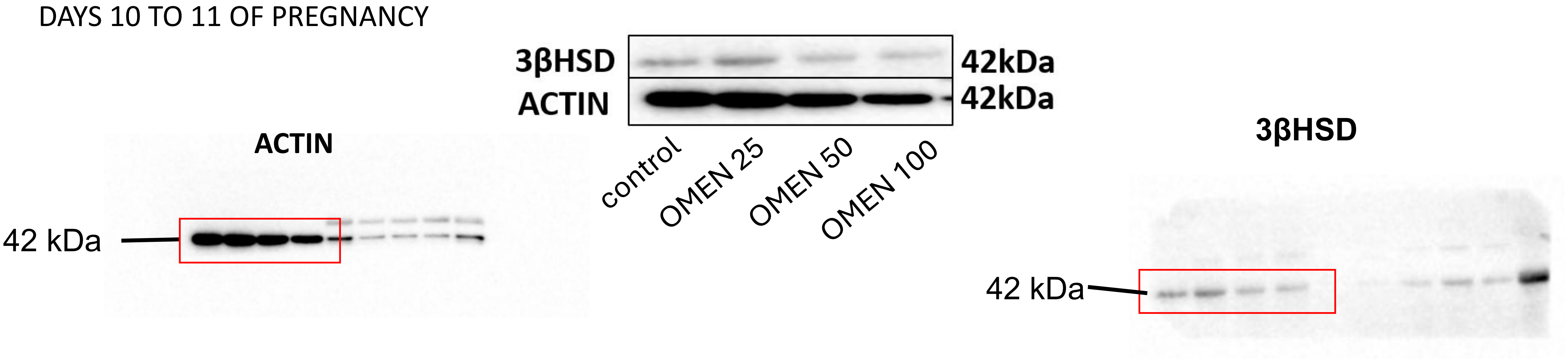

Molecular marker: Thermo Scientific™ PageRuler™
Prestained Protein Ladder, 10 to 180 kDa, 11812124

Figure 2. (L) The protein expression level of 3βHSD in endometrial explants was detected by Western blot (n = 5).

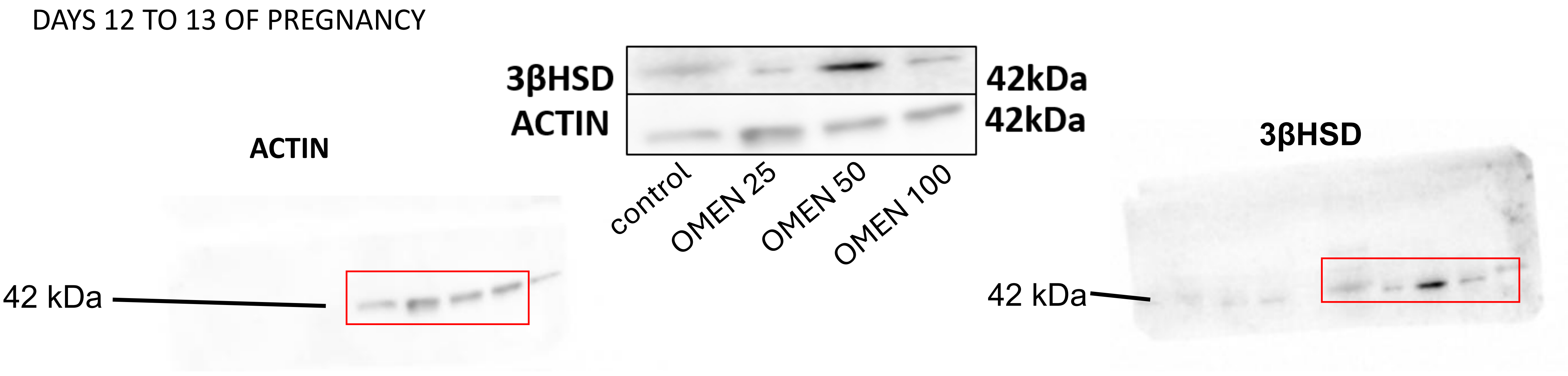

Molecular marker: Thermo Scientific™ PageRuler™
Prestained Protein Ladder, 10 to 180 kDa, 11812124

Figure 2. (M) The protein expression level of 3βHSD in endometrial explants was detected by Western blot (n = 5).

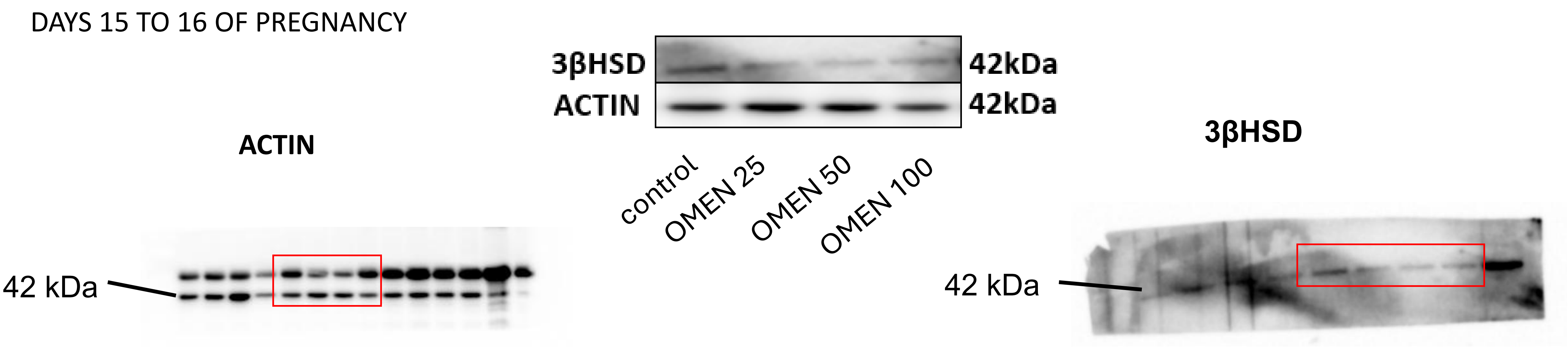

Molecular marker: Thermo Scientific™ PageRuler™
Prestained Protein Ladder, 10 to 180 kDa, 11812124

Figure 2. (N) The protein expression level of 3βHSD in endometrial explants was detected by Western blot (n = 5).

DAYS 27 TO 28 OF PREGNANCY

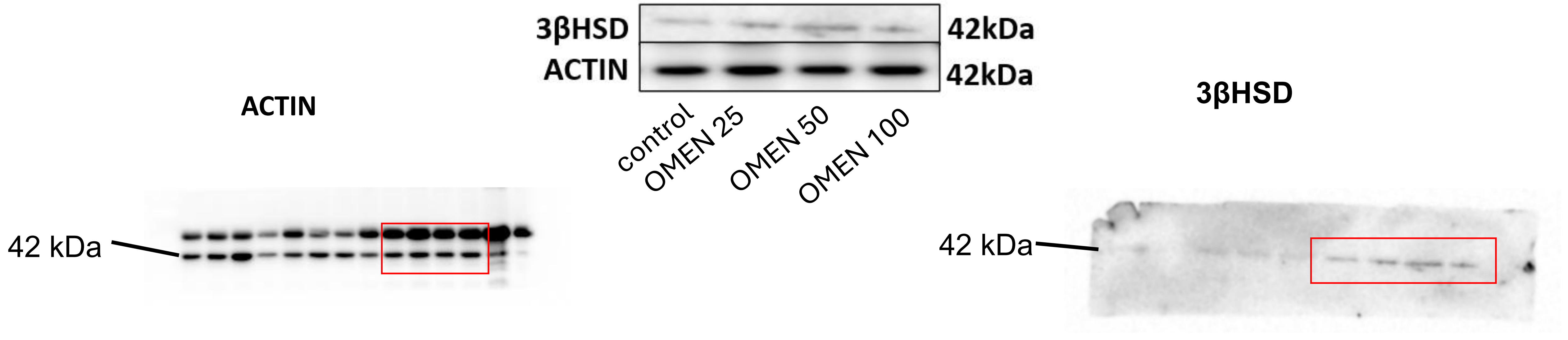

Molecular marker: Thermo Scientific™ PageRuler™
Prestained Protein Ladder, 10 to 180 kDa, 11812124

Figure 2. (O) The protein expression level of 3βHSD in endometrial explants was detected by Western blot (n = 5).

DAYS 10 TO 12 OF THE CYCLE

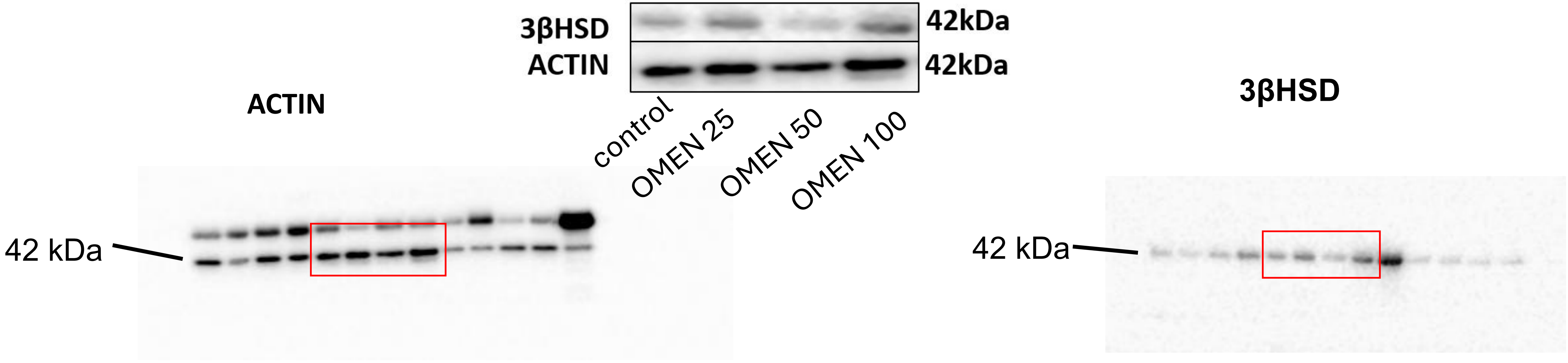

Molecular marker: Thermo Scientific™ PageRuler™
Prestained Protein Ladder, 10 to 180 kDa, 11812124

Figure 3. (A) The protein expression level of $P450_{C17}$ in endometrial explants was detected by Western blot (n = 5).

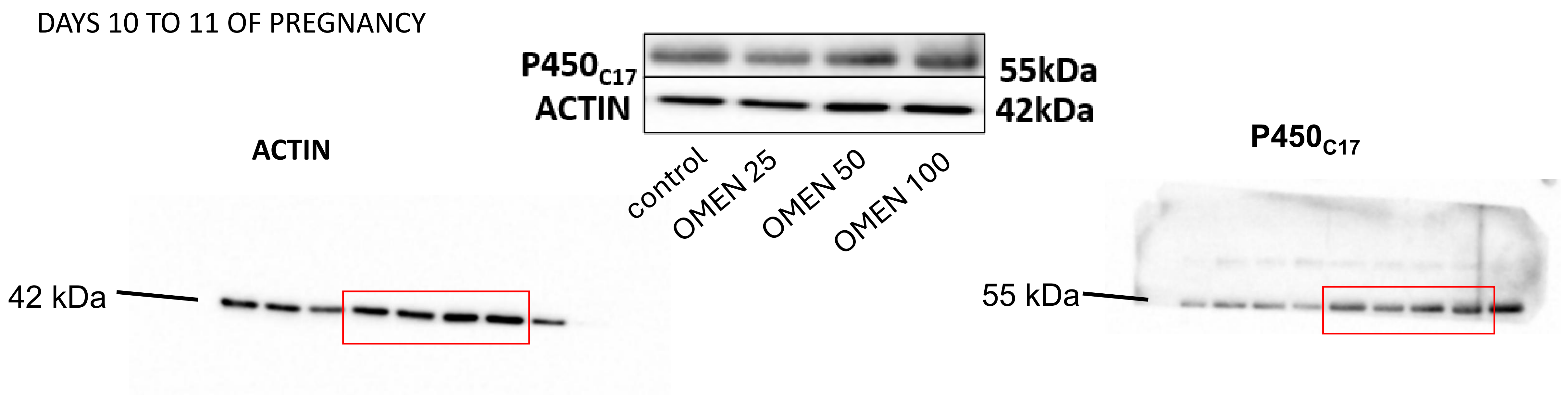

Molecular marker: Thermo Scientific™ PageRuler™
Prestained Protein Ladder, 10 to 180 kDa, 11812124

Figure 3. (B) The protein expression level of $P450_{C17}$ in endometrial explants was detected by Western blot (n = 5).

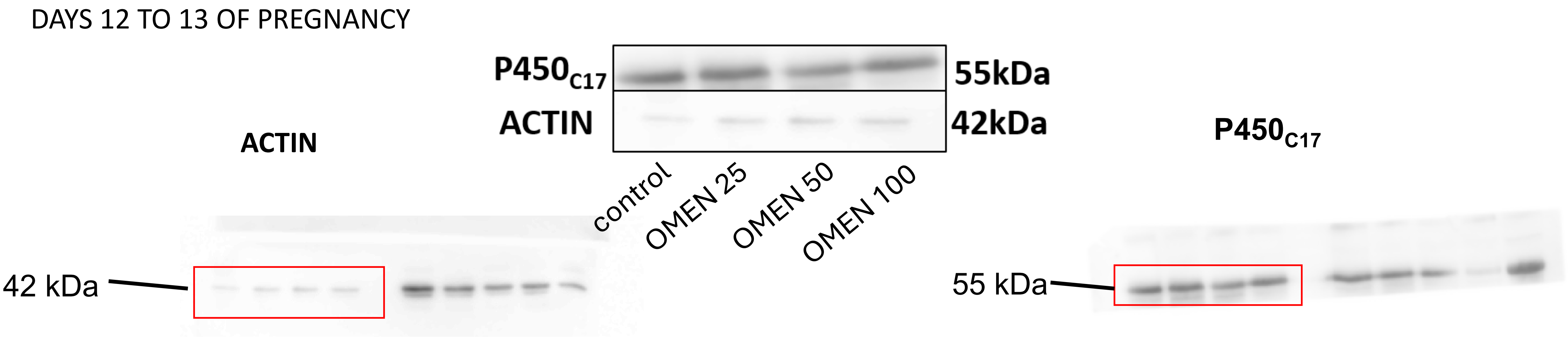

Molecular marker: Thermo Scientific™ PageRuler™
Prestained Protein Ladder, 10 to 180 kDa, 11812124

Figure 3. (C) The protein expression level of $P450_{C17}$ in endometrial explants was detected by Western blot (n = 5).

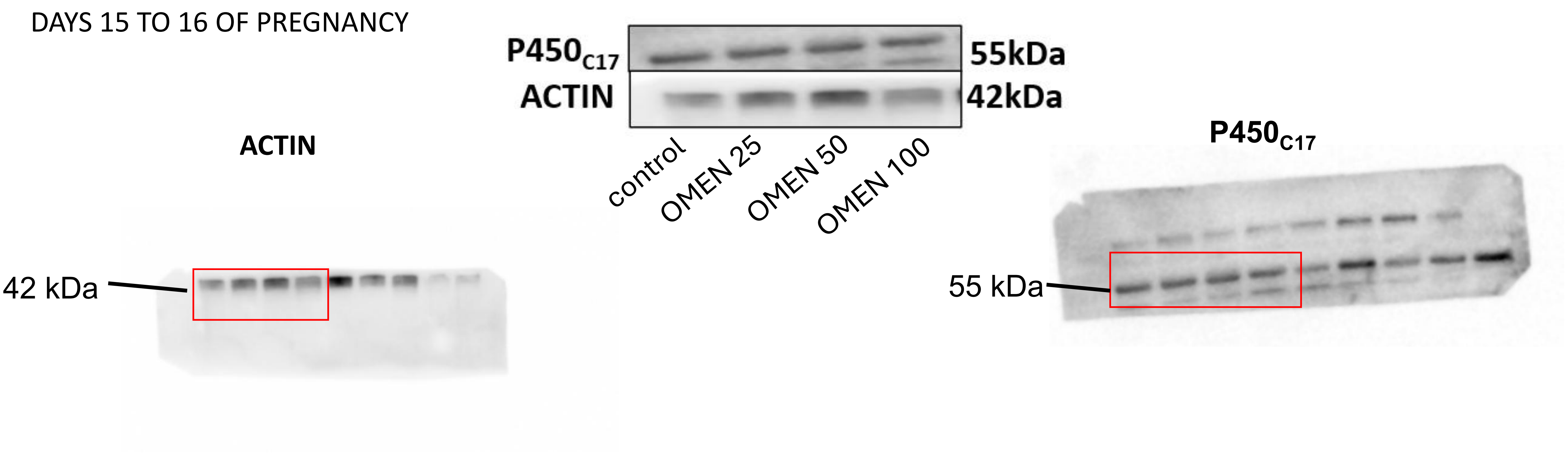

Molecular marker: Thermo Scientific™ PageRuler™
Prestained Protein Ladder, 10 to 180 kDa, 11812124

Figure 3. (D) The protein expression level of $P450_{C17}$ in endometrial explants was detected by Western blot (n = 5).

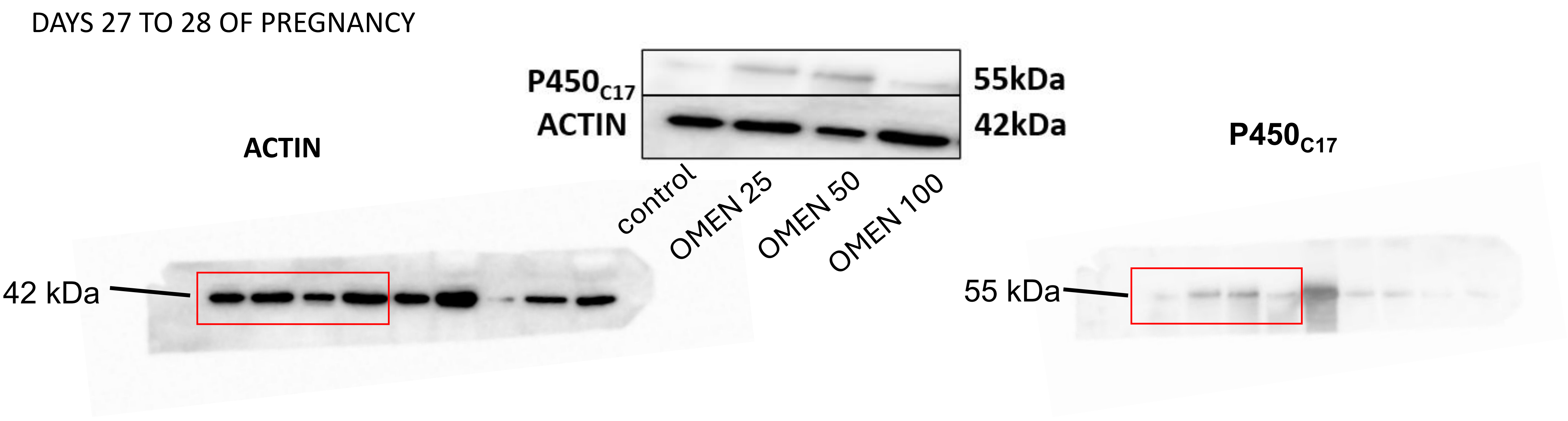

Molecular marker: Thermo Scientific™ PageRuler™
Prestained Protein Ladder, 10 to 180 kDa, 11812124

Figure 3. (E) The protein expression level of $P450_{C17}$ in endometrial explants was detected by Western blot (n = 5).

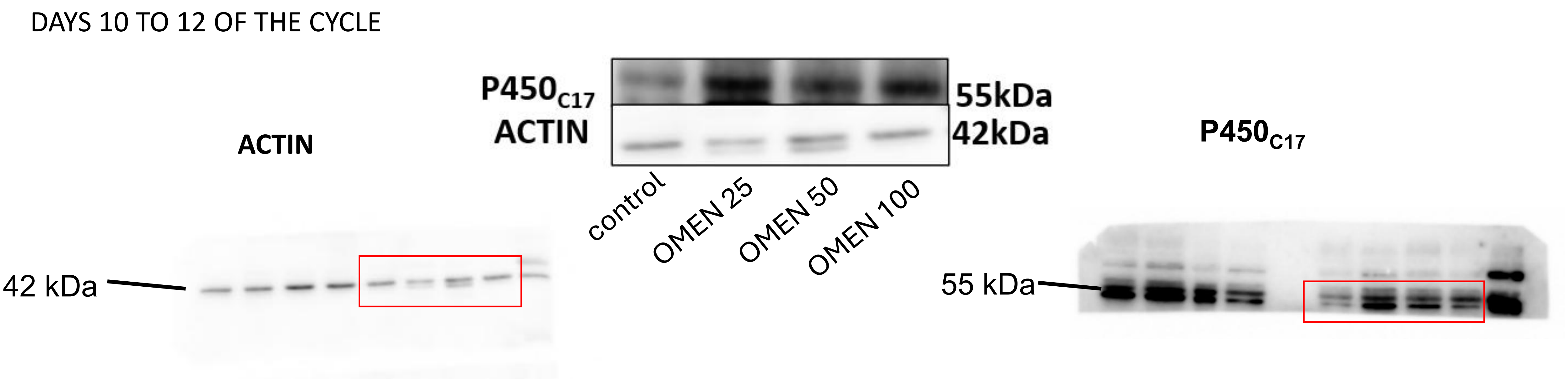

Molecular marker: Thermo Scientific™ PageRuler™
Prestained Protein Ladder, 10 to 180 kDa, 11812124

Figure 3. (F) The protein expression level of $P450_{AROM}$ in endometrial explants was detected by Western blot (n = 5).

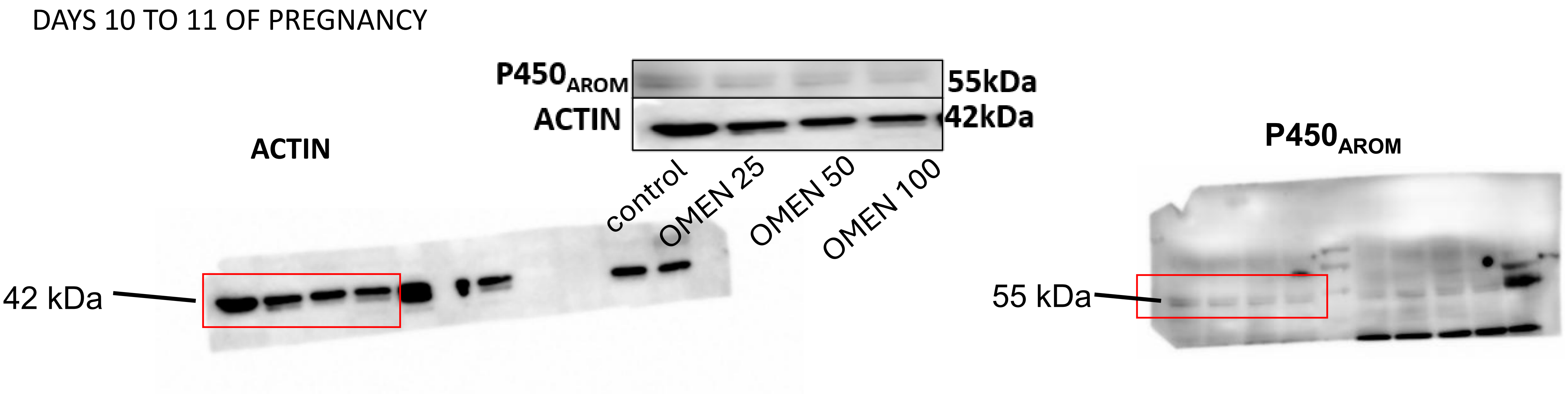

Molecular marker: Thermo Scientific™ PageRuler™
Prestained Protein Ladder, 10 to 180 kDa, 11812124

Figure 3. (G) The protein expression level of $P450_{AROM}$ in endometrial explants was detected by Western blot (n = 5).

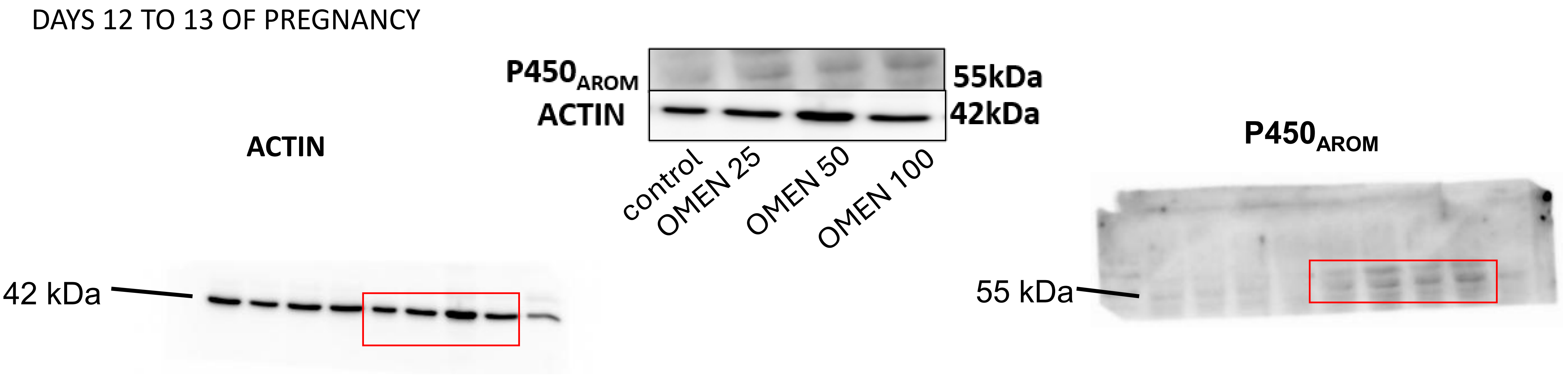

Molecular marker: Thermo Scientific™ PageRuler™
Prestained Protein Ladder, 10 to 180 kDa, 11812124

Figure 3. (H) The protein expression level of $P450_{AROM}$ in endometrial explants was detected by Western blot (n = 5).

DAYS 15 TO 16 OF PREGNANCY

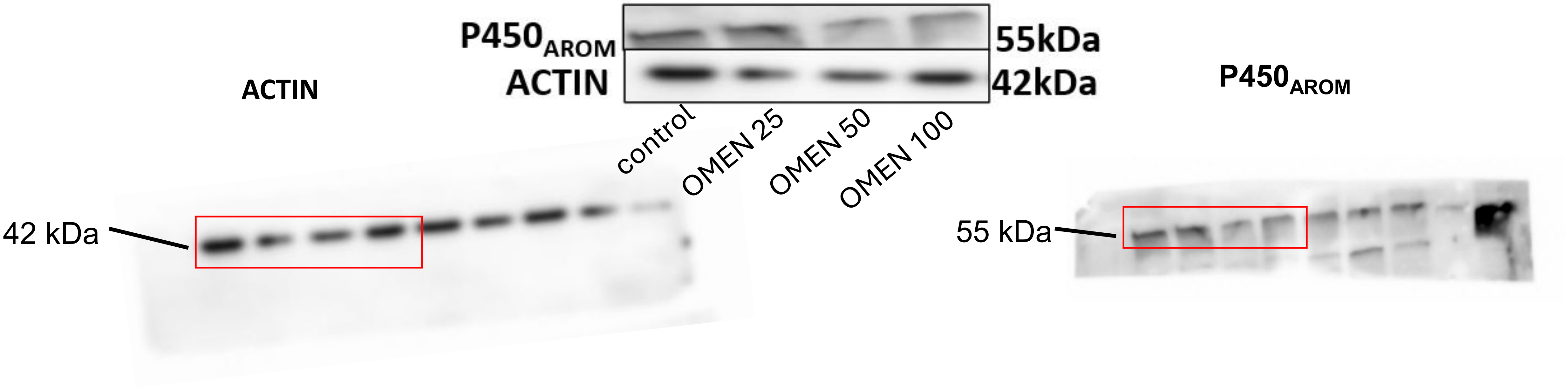

Molecular marker: Thermo Scientific™ PageRuler™
Prestained Protein Ladder, 10 to 180 kDa, 11812124

Figure 3. (I) The protein expression level of $P450_{AROM}$ in endometrial explants was detected by Western blot (n = 5).

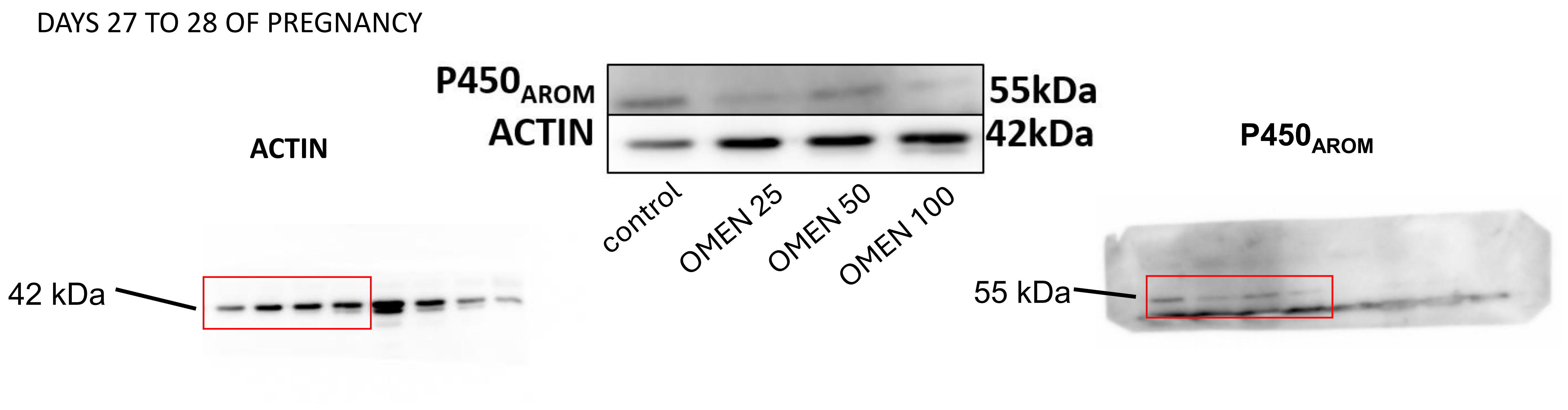

Molecular marker: Thermo Scientific™ PageRuler™
Prestained Protein Ladder, 10 to 180 kDa, 11812124

Figure 3. (J) The protein expression level of $P450_{AROM}$ in endometrial explants was detected by Western blot (n = 5).

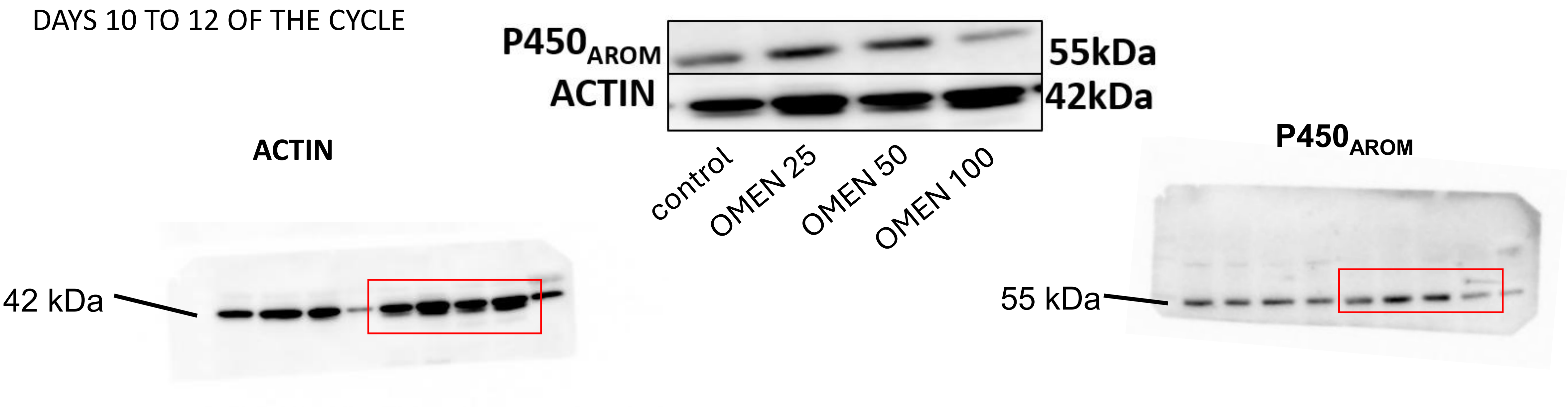

Molecular marker: Thermo Scientific™ PageRuler™
Prestained Protein Ladder, 10 to 180 kDa, 11812124

Figure 4. Phosphorylation of Akt

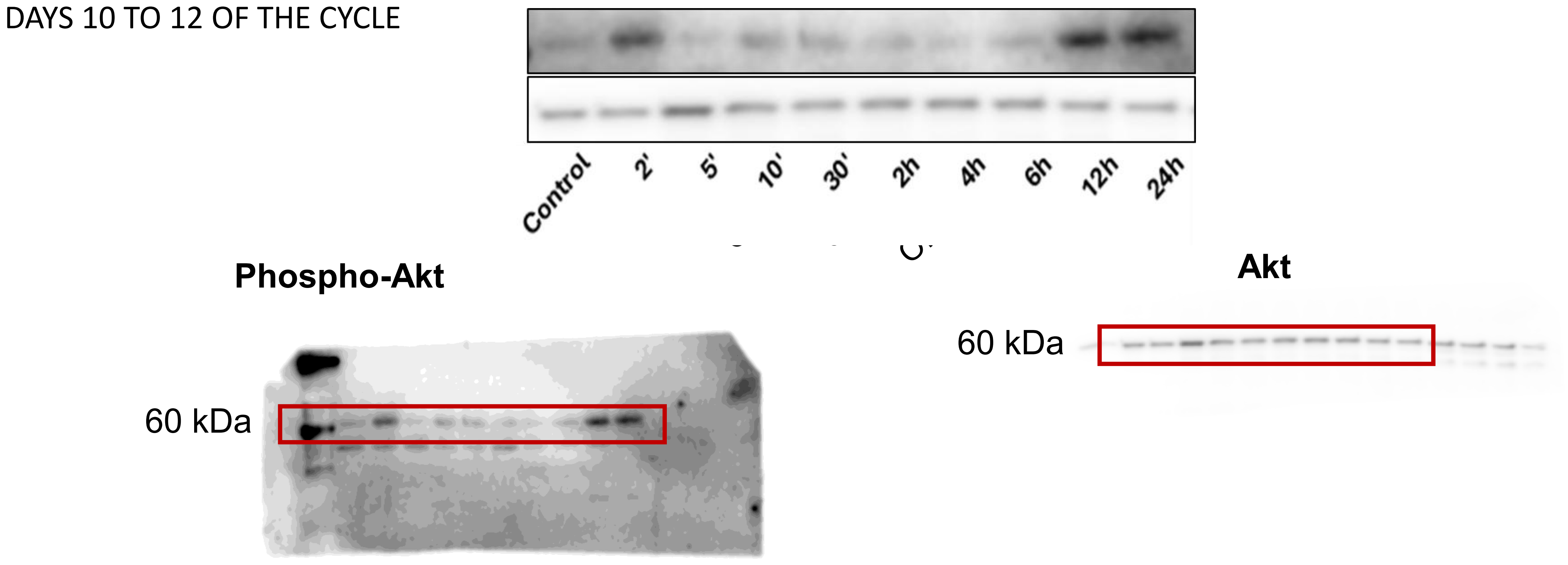

Molecular marker: Thermo Scientific™ PageRuler™
Prestained Protein Ladder, 10 to 180 kDa, 11812124
